# Design and characterization of a polyamine derivative inhibiting the expression of type III secretion system in *Pseudomonas aeruginosa*

**DOI:** 10.1038/srep30949

**Published:** 2016-08-03

**Authors:** Chao Wang, Xiaoling Liu, Jing Wang, Jianuan Zhou, Zining Cui, Lian-Hui Zhang

**Affiliations:** 1 Institute of Molecular and Cell Biology, 61 Biopolis Drive, 138673, Singapore; 2 National Cancer Centre Singapore, 11 Hospital Drive, 169610, Singapore; 3 Chongqing Academy of Chinese Materia Medica, 400065, China; 4 Guangdong Province Key Laboratory of Microbial Signals and Disease Control, South China Agricultural University, 510642, China

## Abstract

The type III secretion system (TTSS) of *Pseudomonas aeruginosa* is a key virulence determinant for infection of eukaryotic hosts. Based on the findings that spermidine-mediated host-pathogen signalling is important for activation of type III secretion systems (TTSS), in this study, we designed, synthesized and evaluated a series of polyamine derivatives for their potentials in inhibiting the expression TTSS in *P. aeruginosa*. *In vitro* assay of 15 compounds synthesized in this study unveiled stringent structural requirements for TTSS-inhibitory activity. Among them, R101SPM, a conjugate between rhodamine 101 and spermine, showed a potent activity in inhibition of the TTSS gene expression and in attenuation of the TTSS-mediated cytotoxicity on human cells. *In vivo* analysis demonstrated that R101SPM could rescue mice from the lethal infection by *P. aeruginosa*. Moreover, genetic analysis showed that the full TTSS-inhibitory activity of R101SPM required a functional spermidine transporter. Taken together, our results present a new class of lead molecules for developing anti-virulence drugs and demonstrate that the spermidine transporter SpuDEGHF of *P. aeruginosa* is a promising drug target.

*Pseudomonas aeruginosa* is an opportunistic human pathogen that causes acute and chronic infections in immunocompromised patients. Infections with *P. aeruginosa* could be fatal for the patients with cystic fibrosis, cancer, AIDS, and severe burns^1^. Due to the multiple-drug resistance mechanisms and large repertoire of virulence factors intrinsic to *P. aeruginosa*, the infections caused by this notorious pathogen is difficult to combat by using conventional bacteriostatic and bactericidal antibiotics^2^,^3^,^4^,^5^. Hospital strains of *P. aeruginosa* that cause untreatable infections have been recently reported^2^. Thus, there is an urgent need for exploring and developing new therapeutic approaches for sustainable control of *P. aeruginosa* infections.

Type III secretion systems (TTSS) are the key virulence determinants of many bacterial pathogens, including *Shigella*, *Salmonella, Chlamydia*, *Yersinia*, *Escherichia* and *Pseudomonas* species^3^,^4^. The systems mediate a complex membrane transport process that directly delivers bacterial effector proteins from bacterial cytoplasm into eukaryotic cell cytoplasm to enable bacterial infections. *P. aeruginosa* contains one set of TTSS system, which translocates four bacterial effectors into host cells to facilitate establishing and disseminating acute infections. Functional TTSS of *P. aeruginosa* is strongly associated with poor clinical outcomes in patients with lower-respiratory and systemic infections^5^. Genetic inactivation of TTSS significantly attenuated the virulence of *P. aeruginosa*^6^,^7^,^8^. In the *P. aeruginosa* strain PAO1, the genes required for the TTSS apparatus are clustered in a 12-kb region of the chromosome, where the *psc* and *prc* genes primarily encode the TTSS structural components, and the *exs* and *pop* genes encode the regulatory proteins. However, the genes encoding TTSS effectors, namely the *exo* genes, are separately located on the chromosome. To date, four *exo* genes (*exoS*, *exoU*, *exoT* and *exoY*) have been identified in *P. aeruginosa* whose products are actively transferred into the host cells. ExoS and ExoT are ADP-ribosyltransferase and possess the GTPase-activating activity. Once translocated, both effectors perturb the host Rac signalling system and cause cell apoptosis/death^6^,^7^,^9^,^10^. On the other hand, ExoU is a lipase and ExoY is an adenylate cyclase, both affecting the host cAMP-Ca^2+^ signalling mechanism and consequently inhibiting the host defence system^8^,^11^,^12^. By injecting these effectors, TTSS serves as a key virulent determinant of *P. aeruginosa* for successful infection of mammalian hosts.

TTSS is regulated by environmental conditions. Host contact and low calcium are two potent inducing signals, and metabolic stress, DNA damage, high concentrations of extracellular Cu^2+^, and low osmolarity have also been implicated as repressive signals^13^. However, all of these signals are eventually converged to the ExsA protein. ExsA is a member of the AraC family transcriptional activators with a DNA-binding domain at C-terminus, which induces the TTSS transcription by directly binding to a consensus sequence located at the upstream of all the TTSS operons^14^,^15^,^16^. In the genome of *P. aeruginosa*, *exsA* forms a single transcription unit with *exsC*, *exsE* and *exsB*^13^. Genetic knockout of *exsA* abrogates the TTSS apparatus and attenuates the infectivity of *P. aeruginosa*^5^. Recently, we found that the polyamine molecules from mammalian hosts, including spermidine and spermine, act as key signals to induce TTSS expression. Knockout of the spermidine-specific transporter SpuDEFGH specifically decreased the TTSS gene expression and drastically attenuated the TTSS-mediated cytotoxicity of *P. aeruginosa*. Exogenous addition of spermidine triggered the expression of TTSS in an ExsA-dependent manner^17^, indicating a critical role of these polyamine signals in activating TTSS and facilitating *P. aeruginosa* infection.

The spermidine transporter of *P. aeruginosa* contains five proteins, i.e., the periplasmic substrate-binding proteins SpuD and SpuE, the ATPase SpuF, and the inner membrane permeases SpuG and SpuH that constitute the cross-membrane channel^18^. Our more recent study showed that SpuD is a putrescine-preferential binding protein, andSpuE is a spermidine-specific binding protein^19^. Comparison of the free form and the spermidine-binding form demonstrated the substrate-binding SpuE undergo an “open-to-closed” conformational switch with the resultant closed ligand-bound form. Similar to the maltose transporter system which have been well characterized^20^,^21^, the SpuE-spermidine closed conformation is switched to open form upon binding to the SpuG/SpuH channel and subsequent induction by ATP binding to SpuF, allowing the release of the bound spermidine to the SpuG/SpuH channel^19^.

Given the vital role of SpuDEGHF in transportation of and it ultrahigh affinity to polyamine signals^18^, we thought it might be possible to paralyse pathogen-host communication through synthesis of the transporter-targeting chemical inhibitors. In this study, we designed, synthesized, and biologically evaluated a series of polyamine derivatives on their inhibitory activity against the expression of *P. aeruginosa* TTSS. The results unveiled useful clues on the structure-activity requirement for TTSS inhibition, and identified a spermine conjugate with a potent activity in inhibition of the transcription of TTSS genes of *P. aeruginosa*. Our data indicate that this compound represents a novel class of lead molecules for developing new antibacterial and anti-virulence agents, which have potentials to combat the diseases mediated by *P. aeruginosa*.

## Results

### Characterization of the spermidine-regulated TTSS expression

**Figure 1:**
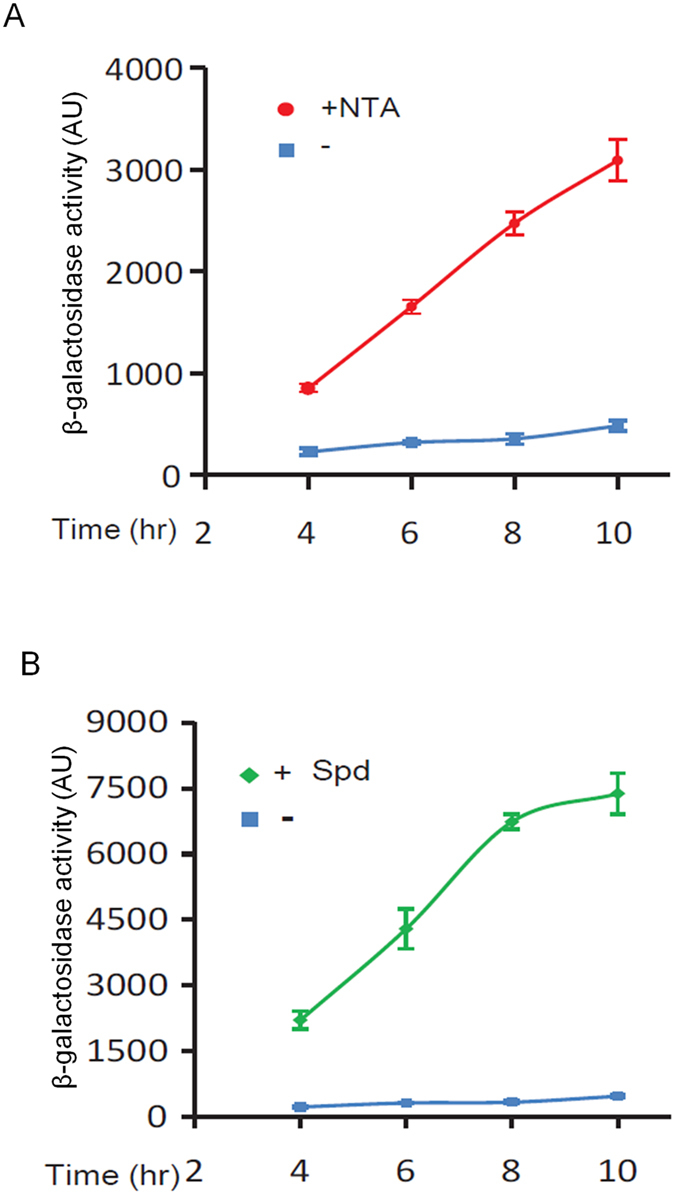
Characterization of the spermidine-induced TTSS regulation. (**A**) NTA induced the β-galactosidase activity of the PCZ reporter strain. Overnight PCZ culture was freshly inoculated with a 0.1% dilution in LB medium (blue square) or LB medium supplemented with 7.5 mM NTA (red circle); samples were collected at the specified time points for β-galactosidase activity assay. (**B**) Spermidine (Spd) stimulation of TTSS was independent of calcium depletion. Overnight cell culture of strain PCZ was inoculated at 1:1000 ratio in the CaCl_2_-containing mininal medium (MM) (blue square) or in the CaCl_2_-containing MM medium supplemented with Spd (0.7 mM) (green diamond).

To facilitate examination of the TTSS-inhibitory activity of synthesized compounds, we established a cell-based assay system using a reporter strain PCZ. The PCZ strain was generated by fusing the *exsCEBA* promoter into the mini-CTX-lacZ vector and integrating into the genome of *P. aeruginosa* strain PAO1^17^. Thereby, PCZ carries one copy of *lacZ* whose transcription is driven by the *exsCEBA* promoter. We firstly evaluated the β-galactosidase activity of this reporter strain under various conditions, and found the enzyme activity was dramatically enhanced in the presence of NTA, which is a Ca^2+^ chelator commonly used to induce TTSS expression in laboratories. As shown in Fig. 1A, while PCZ exhibited a basal level of β-galactosidase activity in Luria-Bertani (LB) medium, NTA notably increased the enzyme activity in a time-dependent manner. These results are consistent with previous results that TTSS is induced by Ca^2+^ depletion, suggesting that the the *exsCEBA* promoter-driven β-galactosidase activity of PCZ could genuinely reflect the TTSS expression in strain PAO1.

We then examined the responsiveness of PCZ to exogenous addition of spermidine molecules, one of the necessary considerations when developing spermidine-derived TTSS inhibitors. Given that the LB medium itself contains substantial amount of spermidine that might complicate interpretation of results, we used minimal medium (MM) in this experiment. As shown in Fig. 1B, addition of spermidine in MM medium dramatically enhanced the β-galactosidase activity of PCZ, by at least 20 times at 8 hr post inoculation compared to the blank control. This enhancement was also evident against that induced by NTA alone (~7 times at 8 hr, Fig. 1A), suggesting that spermidine and NTA act synergistically rather than additively on the TTSS transcription. Furthermore, this result suggests that regulation of TTSS by spermidine is independent of the signalling pathway for Ca^2+^-depletion^22^, suggesting the spermidine signalling system could serve as a new target for developing TTSS inhibitors in *P. aeruginosa*.

### Chemical synthesis and bioassay of spermine conjugates

**Figure 2:**
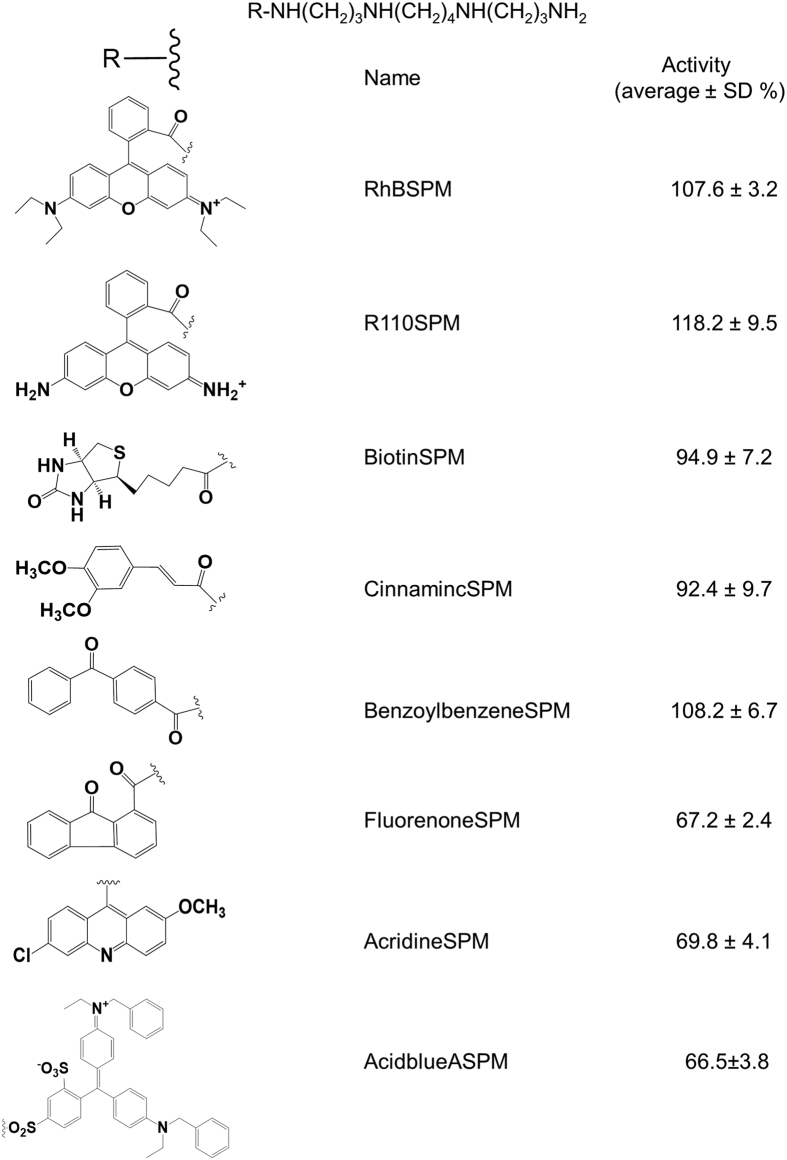
Summarized inhibitory activity of spermine conjugates on TTSS in *P. aeruginosa*. Overnight grown culture was diluted with a ratio of 1:1000 in LB supplemented with specified compounds. Final concentration of the compounds were 50 μM except that R101SPM, R101SPD and RBSPM, whose were 20 μM in the medium. After 4 hr shaking at 37 °C, cells were harvested and washed with PBS once prior to the β-galactosidase activity assay. Culture in absence of compounds was included as controls and the activity (average ± SD) was presented as the percentage of β-galactosidase activity for tested compounds relative to the control. All experiments were repeated three times with duplicates.

The SpuE of the SpuDEGHF transporter interacts with spermidine in high affinity with dissociation constant (Kd) at 1.4 × 10^−8^ M. Ligand binding and release by SpuE appear through an “open-closed-open” mechanism^19^. To exploit these properties and characteristics, we intended to synthesize spermidine analogues by using spermine as the start material to link to compounds with rigid or bulky structure at one of its terminal amino groups. In this way, the conjugates would still contain a functional “spermidine” group, which can be recognized by SpuDEGHF transporter and hopefully, block the signal cross-membrane transportation by paralysing the “open-closed-open” mechanism of SpuE. Figure 2 listed the 9 spermine conjugates synthesized in this study, and Figure S1 illustrated the chemical synthesis route, HPLC purification, NMR and mass spectrometry characterization by using R101SPM, the rhodamine 101 (R101) conjugate of spermine, as an example. Briefly, conjugation of rhodamine 101 with spermine was catalysed by 1.1′-carbonyldiimidazole in dimethylformamide (DMF) solution (Figure S1A). Using the preparative SymmetryPrep^TM^ C18 reverse-phase column (7 μM, 7.8 × 150 mm, Waters), the substrate rhodamine 101 was eluted with a retention time at 13 min in the HPLC profile, and the crude reaction product of R101SPM was purified from the fraction at 5.5 min. On the analytic reverse-phase HPLC column, this purified compound showed a single UV peak at 9.6 min (Figure S1B). The high resolution mass spectrometry analyses showed the measured molecular weight of this purified compound as 675.4402, which was comparable with the theoretical 675.4381 of R101SPM. MS-MS results were in agreement with the expected formula of R101SPM as C_42_H_55_O_2_N_6_ (Figure S1C), where the fragment of 964.9 could be explained as 675 (R101SPM) + 114(TFA) + 114(TFA) + 23(Na) + 39(K). Furthermore, NMR was implemented to dissect the chemical structure of this compound. As given in Figure S1D, the ^1^H NMR results were compatible with that expected for R101SPM, and our not-shown data of ^13^C NMR analyses also validated that the product at 5 min possessed a chemical structure identical to that of R101SPM as proposed (Figure S1A).

All the 9 spermine conjugates were similarly synthesized, purified and characterized as described above. Bioassay results showed that R101SPM displayed the strongest inhibitory activity against the TTSS expression of *P. aeriginosa*, followed by AcidblueSPM, FluorenoneSPM and AcridineSPM, which are acid blue A, fluorenone and acridine conjugates of spermine, respectively, whereas the remaining 5 compounds had negligible activities (Fig. 2).

### Effect of polyamine chain length and substitution on the TTSS inhibitory activity of R101SPM

**Figure 3:**
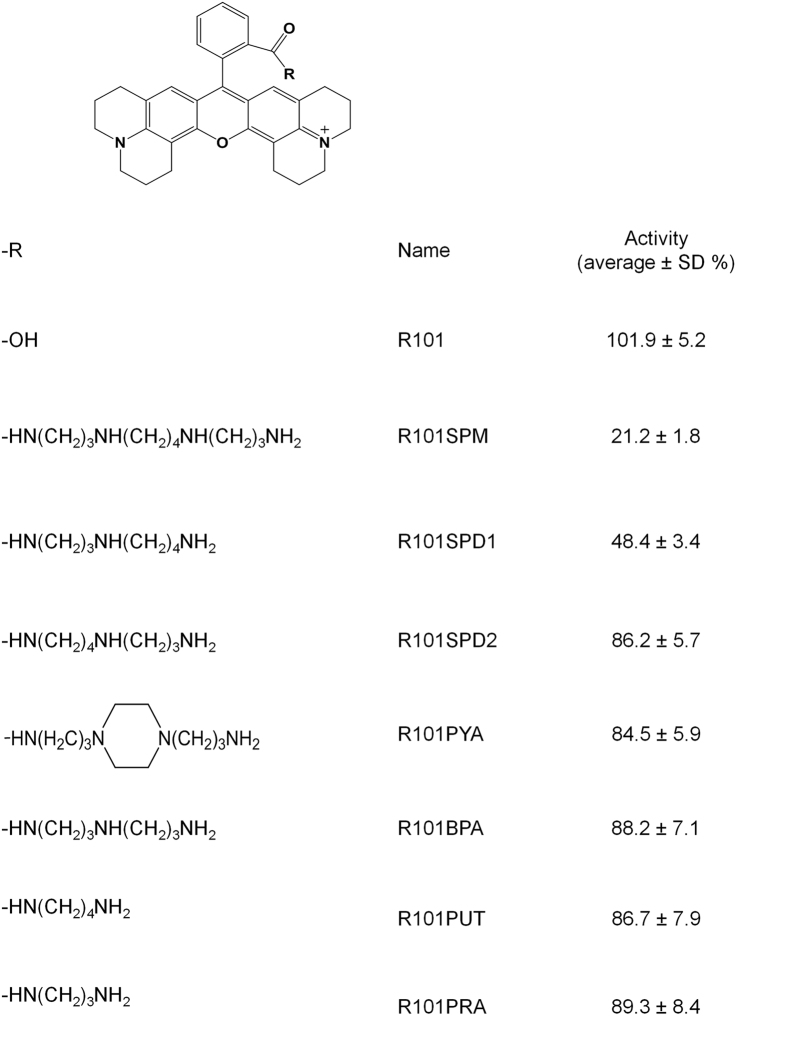
Summarized inhibitory activity of rhodamine 101 derivatives on TTSS in *P. aeruginosa*. The bioassay was conducted as described in Fig. 2. The inhibitory activity (average ± SD) was presented as the percentage of β-galactosidase activity for tested compounds relative to the control. All experiments were repeated three times with duplicates.

Given that rhodamine 101-spermine conjugate R101SPM showing a promising activity on inhibition of TTSS expression, we set to change the length and substitution of spermine to explore the structure-activity relationships of R101SPM. We synthesized other 6 rhodamine-polyamine conjugates following the experimental approaches described in the previous section, and tested their inhibitory activities on the TTSS expression of *P. aeruginosa* with R101SPM and rhodamine 101 (R101) included as controls. Results showed that substitution of spermine with spermidine caused over 50% decrease in its TTSS-inhibitory potency (Fig. 3). Replacement of spermine with other polyamines carrying various methylene groups or substitution almost abolished the inhibitory activity (Fig. 3), suggesting that the “-CONH-(CH_2_)_3_-NH-(CH_2_)_4_-” unit is an important determinant for the inhibitory activity of R101SPM.

### Characterization of the inhibitory properties of R101SPM

**Figure 4:**
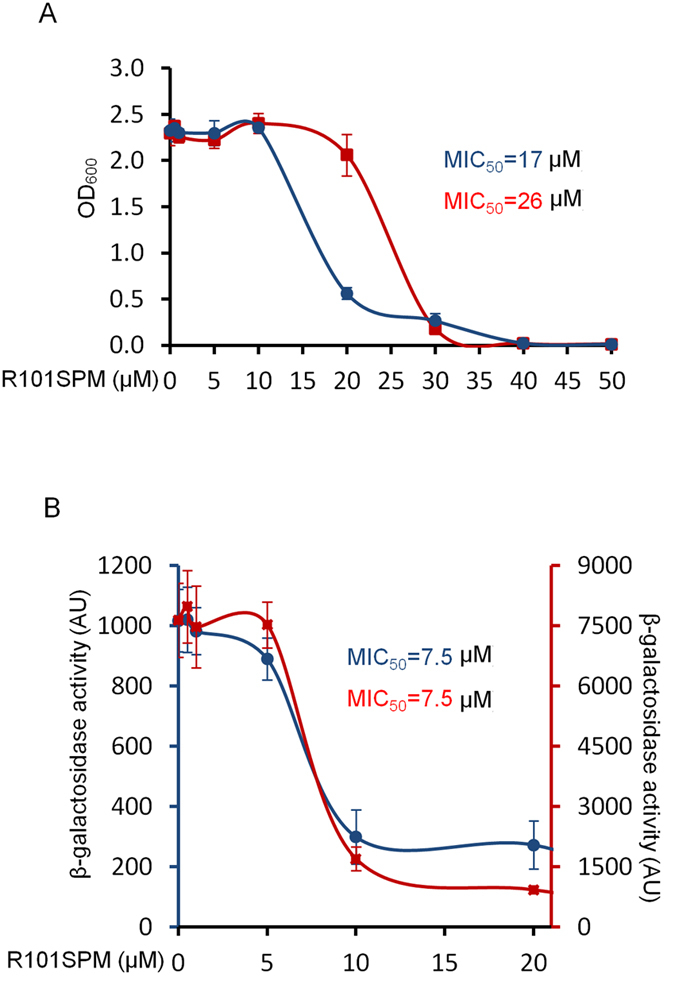
Evaluation of the R101SPM inhibitory effect on *P. aeruginosa*. (**A**) Determination of MIC_50_ for growth inhibition of strain PCZ. Fresh PCZ cells were inoculated with the initial OD_600_ of 0.03 in LB medium (blue circle) or LB medium containing 0.7mM spermidine (red square). R101SPM was added as indicated, and the cells were grown at 37 °C with shaking at 250 rpm for overnight. Bacterial growth was measured by reading OD_600_. (**B**) Determination of MIC_50_ for TTSS inhibition of PCZ. Overnight cells were inoculated with 0.1% dilution in LB medium (blue circle, left y-axis) or LB medium containing 0.7 mM spermidine (red square, right y-axis). Samples were collected at 4^th^ hour post inoculation for the β-galactosidase activity assay.

R101SPM was examined for its inhibitory activity against bacterial growth and TTSS expression. In LB medium, we found that R101SPM effectively inhibited the growth of *P. aeruginosa*, with a MIC_50_ of 17 μM. This inhibition, however, was relieved by supplementation of 0.7 mM spermidine in the medium, with MIC_50_ being increased up to 26 μM (Fig. 4A). The relief of R101SPM-mediated growth inhibition was in a dose-dependent manner but only within a concentration range as higher concentrations of spermidine appeared toxic to bacterial cells under the conditions used in this study. Polyamines including spermidine play multifaceted roles in bacterial cells, and the mechanism underlying this relief is currently not clear. One possible explanation that spermidine cannot fully recover the bacterial growth as seen this experiment is that R101SPM, like spermidne, inhibits bacterial growth by multiple mechanisms. Inactivation of SpuE by physical binding could be one of them, although further investigations are required to strengthen the mechanistic understanding. Nevertheless, relief of R101SPM inhibition by spermidine indicates that both molecules may act on the same target(s) essential for the growth of *P. aeruginosa*, and suggests a promising potential of R101SPM in antagonizing the biological functions of spermidine.

Using the PCZ reporter strain, we assessed the inhibitory activity of R101SPM on the TTSS expression. As expected, this compound exhibited a potent activity in inhibition of TTSS, as monitored by the expression of β-galactosidase. The MIC_50_ was determined to be 7.5 μM in the LB medium (Fig. 4B). When supplemented with spermidine, unexpectedly, inhibition of TTSS expression by R101SPM was not significantly relieved (Fig. 4B), different from that on bacterial growth (Fig. 4A). The mechanisms accounting for this intriguing difference are currently unknown, but it is rational to speculate that R101SPM employs different mechanisms to inhibit bacterial growth and suppress TTSS expression. Significantly, these results indicate that the TTSS inhibition mediated by R101SPM should not be ascribed to its retardation or detrimental effect on bacterial growth.

### Validation of inhibitory effect of R101SPM on TTSS expression

**Figure 5:**
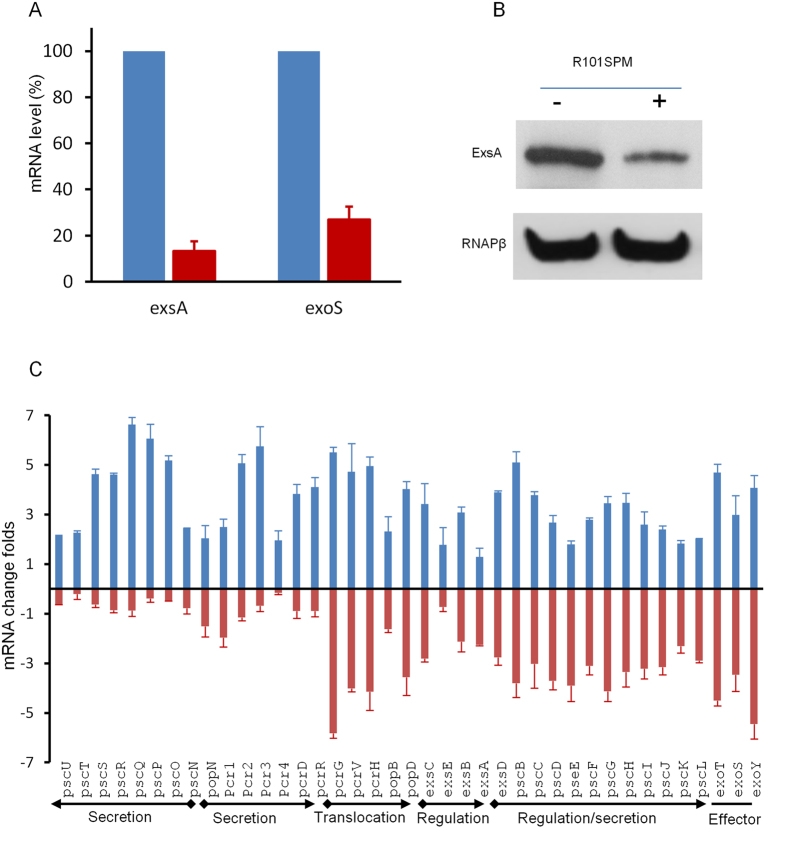
Validation of R101SPM inhibition on TTSS of *P. aeruginosa*. (**A**) Real time qRT-PCR analyses for the transcriptions of *exsA* and *exoS*. Fresh PA01 cells were cultured in MINS medium with 0.7mM spermidine (Spd) in absence (blue column) or presence of 20 μM R101SPM (R101M)(red column). Total RNAs were extracted when the OD_600_ of cultures reached 1.0 and the transcript levels were analysed by real time RT-PCR. Samples without R101SPM treatment was included as control and its mRNA levels were arbitrarily set as 100%. (**B**) Western blot detection of ExsA and RNAPβ in LB + NTA media. Overnight bacterial cultures were inoculated at a 1:1000 ratio to fresh LB plus NTA with or without R101SPM (20 μM). After OD_600_ reaching 1.0, 1 ml of cell cultures were collected and samples prepared by boiling for 5 minutes and separated by 10% SDS-PAGE. Western blot analysis was performed following the standard protocols using antibodies against ExsA and RNAPβ (RNA polymerase β subunit). (**C**) Microarray analysis of the TTSS gene expression in PAO1 treated with spermidine and R101SPM. Fresh PA01 cells were cultured in MINS with 0.7 mM spermidine in absence (blue column) or presence of 10 μM R101SPM (red column). When OD_600_ reached 1.0, total RNAs were extracted, reverse transcribed, fragmented and labeled as suggested. The processed RNAs were hybridized to Affymetrix GeneChip *P. aeruginosa* microarrays, and the chips were washed, scanned and analysed as described in text.

Besides monitoring the β-galactosidase activity of PCZ, we also validated the inhibitory effect of R101SPM by directly examining the transcripts and translational products of TTSS genes in the wild type PAO1 strain. We firstly used qRT-PCR to quantify the changes in the mRNA levels of *exsA* and *exoS* in the presence and absence of R101SPM. ExsA is a master transcriptional activator for TTSS expression and ExoS is one of the TTSS effectors. As shown in Fig. 5A, R101SPM treatment dramatically decreased the mRNA level of *exsA* by more than 85% relative to the untreated control. Similarly, a comparable decrease was observed for the *exoS* gene (Fig. 5A). Agreeable with the results, western blotting analysis showed that the protein level of ExsA was substantially reduced by R101SPM treatment (Fig. 5B).

To determine the expression levels of other TTSS genes upon R101SPM treatment, we further conducted microarray analysis. In total, 156 genes were up-regulated and 191 genes down-regulated upon R101SPM treatment (Tables S1 and S2). The majority of these genes were hypothetic proteins with uncharacterized functions, including 51 being up-regulated and 70 down-regulated. Among these genes,only a few are associated with cellular metabolism, quorum sensing and bacteria growth. Strikingly, all the 39 spermidine-induced TTSS genes appeared down-regulated with 22 genes being significantly inhibited by R101SPM (≥2-fold). The R101SPM significantly inhibited genes include *exsA* and *exoS,* agreeable with the qRT-PCR data, and other genes encoding translocation, regulation and TTSS effectors (Fig. 5C). Unambiguously, these microarray results confirmed that R101SPM is capable of inhibiting the transcription of TTSS and thus could disable assembly of a functional TTSS system.

### R101SPM attenuates the TTSS-mediated cytotoxicity and bacterial virulence

**Figure 6:**
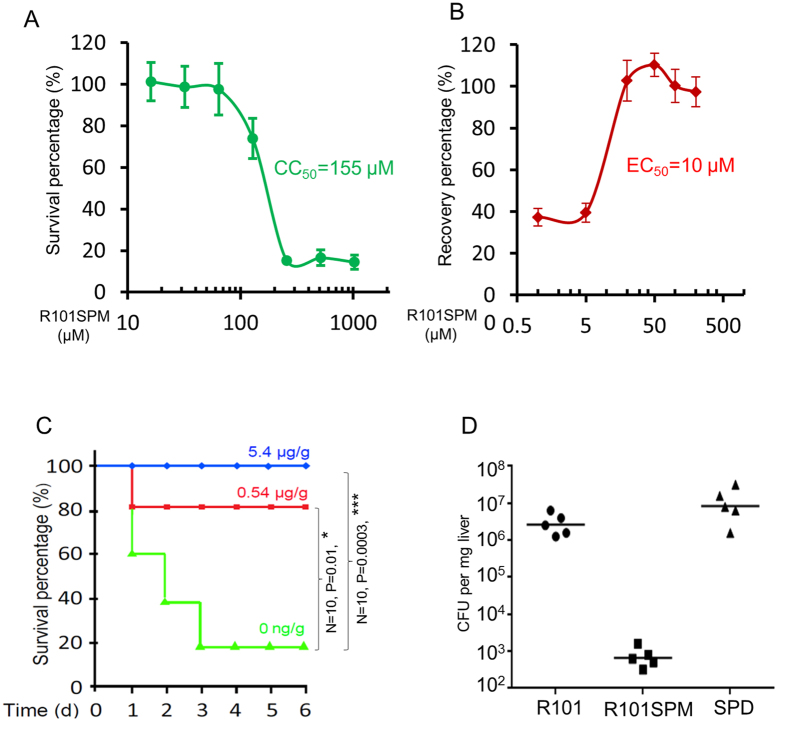
Valuation of R101SPM effect on human cells and mouse animals. (**A**) Determination of the cytotoxicity of R101SPM to Hela cells. Hela cells were seeded at 5000 cells per well in 96-well plate in DMEM supplied with 10% FBS (Invitrogen). After culture overnight, the cells were washed once with PBS and incubated with FBS-free medium supplemented with various concentrations of R101SPM as indicated. With 4-hour incubation, the survival cells were assayed with the Cell Counting Kit-8 (Dojindo) and the survival percentage of cells without R101SPM treatmentwas arbitrarily set as 100% as a control. (**B**) Determination of effective concentration of R101SPM for rescuing Hela cells from TTSS-mediated cytotoxicity of PAO1. FBS-challenged Hela cells were mixed with PAO1 at an MOI of 20 in presence of R101SPM. After 4 hr co-culturing, the survival cells were assayed with the Cell Counting Kit-8 (Dojindo) and the recovery percentage of cells without PAO1 was arbitrarily set as 100% as a control. (**C**) Protection of mice by R101SPM. The animals were infected with *P. aeruginosa* by intratracheal injection,while R101SPM was administrated by intraperitoneal injection. (**D**) R101SPM reduced bacterial load in mouse liver. Mice were infected with PAO1 together with rhodamine 101 (R101), spermidine (SPD) and R101SPM, respectively. Infected mice were scarified one day post of infection, and livers were sterile homogenized for CFU counting. Data presented were the CFU numbers normalized against the wet weight of livers from five mice. The median was marked with a horizontal line.

Is inhibition of TTSS transcription by R101SPM sufficient to disarm the biological function of TTSS? To answer this question, we first evaluated its cytotoxicity to human cells and then studied its potency in protection of human cells from infection by *P. aeruginosa*. To determine CC_50_ of R101SPM, Hela cells were exposed with various concentrations of R101SPM in serum-free medium, and the cell viability was measured using CCK8 kit (Dojido Molecular Tech, Japan), and the CC_50_ value was calculated as the concentration that inhibits 50% of the conversion of WST-8 to formazan by using the GraphPad Prism software. With 2-day incubation, we determined the CC_50_ of R101SPM as25 μM. With a 4-hour incubation, which was used for the EC_50_ assay in Fig. 6B, the CC_50_ of R101SPM was determined up to 155 μM (Fig. 6A). Similarly, we challenged the Hela cells with *P. aeruginosa* and added R101SPM with different concentrations for recovery. Under this condition, we found that treatment with R101SPM was able to rescue the Hela cells from lethal infection by *P. aeruginosa*, with an EC_50_ at 10 μM (Fig. 6B).

We further investigated the efficacy of R101SPM in prevention of bacterial infection in a mouse model. Following the guidelines of the National Advisory Committee for Laboratory Animal Research and the protocols approved by the Institutional Animal Care and Use Committee (Singapore), we infected mice by intraperitoneal injection of *P. aeruginosa* mixed with R101SPM. The results showed that R101SPM significantly protected mice against *P. aeruginosa* infection, and this protection occurred in a dosage-dependent manner (Fig. 6C). Treatment with R101SPM at a dosage of 0.54 μg·g^−1^ increased the mice survival rate by about 60% compared with the R101SPM-untreated control challenged with *P. aeruginosa*. When R101SPM concentration was increased to 5.4 μg·g^−1^, all the bacteria-challenged mice were fully protected from death (Fig. 6C). As a side-by side control, we found that R101SPM, even at a concentration of 67.5 μg·g^−1^, was not lethal to mice under the same experimental conditions (Figure S2A).

To exclude the possibility that the protection effect of R101SPM is due to its antibiotic-like activity, we then infected mice at one side of mouse belly and injected the compound at the other side of belly. As shown in Figure S2B, we found that R101SPM was capable of protecting mice from lethal infection by *P. aeruginosa* in this treatment approach, but the effect was not as high as that by mix treatment shown in Fig. 6C. To examine whether mix treatment might cause bacterial death, we conducted a recovery dynamic assay after transient treatment of bacteria with the compound. We found that the viability of strain PAO1 cells was not significantly reduced upon treatment with 2 mM of R101SPM for 30 min (Figure S2C), suggesting that the difference in protection efficacy between mix treatment and separate injection approaches is most likely due to the administration approaches rather than the lethal effect of R101SPM. When we infected mice with intratracheal injection and administrated R101SPM through intravenous injection, we found that R101SPM at a dosage as low as 0.2 μg·g^−1^ was effective in protection of mice. Furthermore, we found that *P. aeruginosa* cells were significantly cleared from the mouse livers with administration of R101SPM (Fig. 6D), which is exciting and encouraging when comparing the data of Figure S2C. Cumulatively, these results demonstrate that R101SPM is a potent anti-virulence inhibitor, both *in vitro* and *in vivo*.

### The full TTSS-inhibitory activity of R101SPM requires a functional spermidine transporter

**Figure 7:**
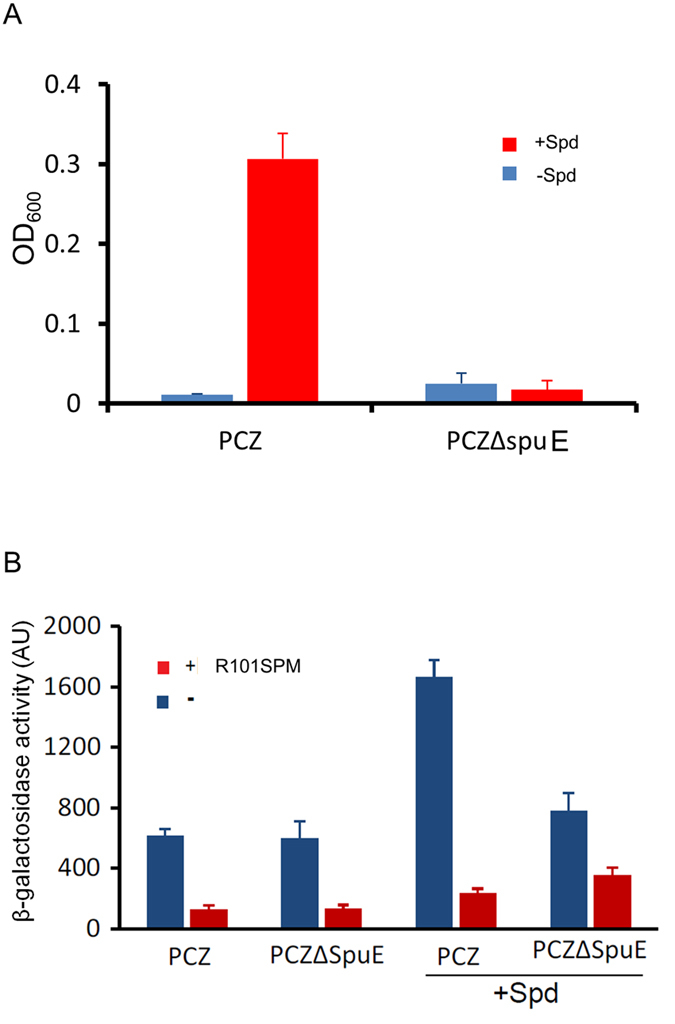
Partial dependence of R101SPM inhibition of TTSS on the spermidine-specific transportation system. (**A**) Dependence of spermidine catabolism on the functional spermidine transporter. Fresh cells were washed three times with PBS and inoculated into the MM medium without carbon and nitrogen source (blue column) or the MM medium with 0.7 mM spermidine (Spd) as the only carbon and nitrogen source (red column). Cell growth was determined by reading OD600 after culture for 16 hr. (**B**) Partial dependence of R101SPM inhibition of TTSS on the functional SpuDEFGH transporter. Overnight cells were inoculated with 0.1% dilution in LB medium or LB medium containing 0.7 mM spermidine, treated with 20 μM R101SPM (red column) or without R101SPM (blue column), respectively. Samples were collected at 4th hour post of inoculation for the β-galactosidase activity assay.

The results in Figs 2 and 3, which showed that polyamine chain length and bulky structural features of spermine conjugates are associated with the potency of compounds in inhibition of bacterial TTSS, suggest that R101SPM may inhibit TTSS expression by blocking spermidine transportation. To address this possibility, we firstly determined whether the SpuDEGHF is the primary transporter of spermidine in *P. aeruginosa*. The results showed the reporter strain PCZ could use spermidine as the sole carbon and nitrogen source to support bacterial growth. In contrast, deletion of *spuE*, which encodes a spermidine binding protein of the transporter, appeared to completely abrogate the transportation of spermidine as the *spuE* mutant could hardly grow in the MM medium with or without spermidine (Fig. 7A).

To test whether the spermidine transportation is required for R101SPM-mediated inhibition of TTSS, we subsequently examined the inhibitory effect of R101SPM on TTSS in the *spuE*-deletion mutant by using the parental strain PCZ as a control. The results showed that in the presence of TTSS-inducer spermidine, the β-galactosidase activity of PCZ was decreased by R101SPM from about 1700 to 250, equivalent to a 6.7-fold reduction, whereas the same enzyme activity of the *spuE* mutant was decreased from about 800 to 350, that is, only a 2.3-fold reduction, by R101SPM treatment (Fig. 7B). Under the same experimental conditions, we found that in the medium without spermidine (under this condition, the SpuDEGHF is not required), R101SPM did not cause obvious difference on the β-galactosidase activity of strain PCZ and the *spuE* mutant, and R101SPM treatment decreased the enzyme activity by about 3-fold compared with the untreated control. Taken together, the results suggest that a functional spermidine transporter is needed for the full activity of R101SPM against the TTSS of *P. aeruginosa,* but the inhibitor could also affect TTSS through an unknown mechanism(s).

### Effect of R101SPM on production of other virulence factors

*P. aeruginosa* deploys a large variety of virulence factors for successful infection, including quorum sensing (QS), biofilm and pyocyanin. To examine the effect of R101SPM on QS, we generated various PAO1 reporter strains where the promoters of *rhlI*, *pqsA* and *lasB* were respectively fused with a promoterless *lacZ* gene. *rhlI* encodes the synthase for producing N-butyryl-L-homoserine lactone, *pqsA* encodes a quinolone synthase and *lasB*, the elastase. These enzymes are either key QS components or the important virulence factor regulated by QS^23^. The β-galactosidase activity assay indicated that addition of spermidine decreased the expression of QS genes *rhlI* and *pqsA*, but treatment with R101SPM did not seem to have any effect on the expression of *rhlI, pqsA* and *lasB* when compared with the untreated control sample (Figure S3A). We also measured the biofilm production in the presence or absence of R101SPM, and found no significant difference between control and treatment (Figure S3B). Pyocyanin is another QS-dependent virulence factor involving in iron metabolism and cytotoxicity, and no significant difference was observed between the R101SPM-treated samples and the untreated control (Figure S3C). Put together, these data are consistent with the notion that R101SPM attenuates the bacterial virulence largely through targeting the TTSS of *P. aeruginosa*.

## Discussions

In this study, a potent TTSS inhibitor R101SPM was identified through rational design and synthesis of polyamine-conjugates based on the mechanical features and characteristics of SpuDEGHF and subsequent TTSS-reporter based drug screening. SpuDEGHF is a spermidine transporter essential for importation of host polyamine signals to trigger the expression of TTSS genes in *P. aeruginosa*^20^. We showed that R101SPM is a potent small molecule inhibitor against the spermidine-induced TTSS gene expression with a MIC_50_ of 7.5 μM (Fig. 4). More importantly, this compound effectively rescued human cells from the bacterial TTSS-mediated cytotoxicity with a EC_50_ of 10 μM, and efficiently protected mice from mortality caused by *P. aeruginosa* infection (Fig. 6). These data suggest that R101SPM may be a promising lead compound for developing anti-TTSS inhibitors to prevent and control of the infections caused by *P. aeruginosa*.

Anti-TTSS inhibitors have been extensively explored as a novel type of agents to combat diseases caused by intractable multidrug-resistant bacteria. A number of small molecule compounds have been screened to disarm TTSS functions in *Chlamydia*, *Shigella*, *Yersinia*, *Salmonella* and uropathogenic *Escherichia coli*, with a purpose to provide a new class of anti-infection drugs in addition to traditional antibiotics^18^,^24^,^25^,^26^,^27^,^28^,^29^. In *P. aeruginosa*, three types of TTSS inhibitors have been reported by screening of chemical libraries commercially available. Among them, two were shown to inactivate the enzyme activity of TTSS effectors ExoU and ExoS, respectively^23^,^30^, and the other one appeared to inhibit the effector secretion and translocation with an unknown mechanism^25^. The results from this study showed that R101SPM may represent a new type of inhibitor acting at the TTSS expression, which appeared primarily act by blocking the spermidine-induced TTSS gene expression. Firstly, the spermidine-induced expression of ExsA, which is the master regulator of *P. aeruginosa* TTSS, was significantly decreased by R101SPM (Fig. 5A,B), Moreover, the anti-TTSS efficacy of R101SPM was decreased by over 4-fold in the absence of a functional spermidine transporter SpuDEGHF (Fig. 7). Furthermore, transcriptional analyses showed that majority of TTSS genes were significantly down-regulated when *P. aeruginosa* was treated with R101SPM (Fig. 5C), whereas production of other virulence factors was not obviously affected by the inhibitor (Figure S3). In this regard, it is interesting to note that functional mutation of the SpuDEGHF transporter also selectively down-regulates the spermidine-induced transcriptional expression of *P. aeruginosa* TTSS genes^20^. Collectively, these findings validate the spermidine transporter SpuDEGHF as a promising drug target for developing of anti-TTSS inhibitors.

The results from this study also provide useful clues on the structure-activity relationship of polyamine conjugates in inhibiting the expression of *P. aeruginosa* TTSS. When comparing the anti-TTSS activity of a range of spermine conjugates, we found that both hydrophobicity and bulky structure appeared important, which is evident as R101SPM and Acidblue ASPM were among the top two showing reasonable activities (Fig. 2). It would be interesting to further test whether binding of these molecules could impede the observed “open-to-closed” switch of SpuE-ligand interaction^19^. In another experiment, when we tested the rhodamine 101 conjugates, we found that the spermine-rhodamine 101 conjugate (R101SPM) showed the best anti-TTSS activity, followed by spermidine-rhodamine 101 conjugates R101SPD1 and R101SPD2 (Fig. 3), suggesting that the polyamine chain length and orientation of the inhibitors could affect their functionality. Low tolerance for alterations in the rhodamine group and the polyamine moiety is somewhat expected given that the transporter SpuDEGHF and its substrate binding protein show a stringent requirement for its substrate^19^,^20^. Further optimization of these two parameters may be able to improve the pharmacologic and toxicological properties of this type of anti-TTSS inhibitors.

Interestingly, two spermine derivatives, i.e., spermatinamine and its derivative pseudoceramine B, isolated from marine sponge, were found capable of inhibiting the effector secretion by the TTSS of *Yerssinia pseudotuberculosis*^30^. The mechanism of TTSS-inhibition has not been reported, and the compounds also displayed strong antibacterial activity. These two compounds are structurally different from R101SPM with both terminal amino groups of spermine fused with bromotyrosine derivatives through amide bonds. While it is hard to predicted at this stage whether these two compounds and R101SPM share similar mechanisms in anti-TTSS and in antibacterial growth, the results of spermatinamine analogues and R101SPM suggest that spermine derivatives are worthy of further investigations in developing new anti-infection agents.

In conclusion, with an initial aim to develop TTSS inhibitors by targeting spermidine signalling pathway, we eventually found a rhodamine-polyamine derivative which inhibits the expression of TTSS in *P. aeruginosa* with a high potency. The full functionality of R101SPM depended on the presence of spermidine transporter and the inhibitor down-regulated the spermidine-induced expression of TTSS genes in *P. aeruginosa*. Our results indicate that blocking spermidine-mediated host-pathogen communication could be a promising approach in developing anti-infection drugs, and show that the spermidine transporter SpuDEGHF is a valid target for drug screening and development.

## Experimental Procedures

### Strains, plasmids and growth conditions

Bacterial strains and DNA plasmids used in this study were listed in Table S3. *Escherichia coli* strains were maintained on Luria-Bertani (LB) agar plates containing antibiotics as necessary (gentamicin, 10 μg/ml; carbenicillin, 150 μg/ml; tetracycline, 5 μg/ml). *P. aeruginosa* strains were maintained on LB medium with antibiotics as necessary (gentamicin, 50 μg/ml; carbenicillin, 300 μg/ml; tetracycline, 100 μg/ml). For TTSS gene expression studies, *P. aeruginosa* strains were cultured in LB supplemented with 7.5 mM (Nitrilotriacetic acid) NTA, or MM medium as indicated^31^. Treatment of small molecule compounds was conducted as described elsewhere. Biofilm formation and pyocyanin production were determined as previously described^32^,^33^,^34^.

### Manipulation of DNA and bacteria

DNA manipulation and molecular cloning were performed by standard methods. The pME-rhlI and pME-lasB plasmids were constructed by inserting PCR-amplified promoters of rhlI and lasB into pME6016, as published^34^. Tn7-pqsA was generated by cloning the pqsA promoter into the mini-Tn7T-Gm-lacZ vector as reported previously^34^. Deletion constructs of pEX18G-spuB and pEX18G-vfr were created with a strategy similar to that used for pEX18G-spuE, which has been published^17^. All plasmids constructed in this study were verified by DNA sequencing with a dye terminator kit and an ABI Prism 3770 sequencer. For bacterial transformation, *E. coli* strains were transformed by heat shock and *P. aeruginosa* strains were transformed by electroporation^35^. Genetic manipulation of *P. aeruginosa* was carried out as previously reported unless otherwise stated. PCZ, the reporter strain for the *exsA* transcription, was derived from *P. aeruginosa* PAO1 by fusing the *exsCEBA* promoter with the promoterless lacZ gene and integrating into the bacterial chromosome^17^. PCZΔvfr, the PCZ strain carrying an in-frame deletion of vfr, and PCZΔspuB, the PCZ strain carrying an in-frame deletion of spuB, together with PCZΔspuE, the PCZ strain carrying the spuE deletion, were generated by allelic exchange as described previously^17^. PAO1(T7T-pqsA), the reporter strain for the pqsA transcription, was generated by integrating the Tn7-pqsA vector into the *att* site of chromosome using published methods^36^.

### Quantification of mRNAs

For total RNAs extraction, fresh PA01 cells were grown in MINS medium supplemented with spermidine (0.7 mM), in absence or presence of R101SPM (10 μM). When the OD_600_ of cultures reached ~1.0, cells were harvested and total RNAs were purified with RNeasy Protect Bacteria Mini Kit (Qiagen). After decontamination by DNase I, the RNAs were qualified by electrophoresis and quantified by Nanodrop 1000 Spectrophotometer. For real time RT-PCR, 0.1 μg total RNAs was used as the template, and the SYBR^®^Green RT-PCR kit (Qiagen) was used to quantify the mRNA levels of target genes on Lightcycler v1.5 (Roche). In parallel, the rpoC gene (encoding the β’ subunit of RNA polymerase) was included as controls. Results were presented as ratios of gene expression between the target gene and the control gene^37^. Real-time RT-PCR primers specific to *exsA* were 5- CGAGCGGAGAATCCTCTATG/5- CG ATGTCGACGATGCTCA, to *exoS* were 5-CAGCGATGAGCAGGGAGT/5-TGGCTATGG CCACTCTGC, and to rpoC were 5-CTGTTCAAGCCGTTCATTTTC/5-CTTGATGGTGG TGGCCATA. For microarray analysis, total RNA samples were prepared as described above for real time RT-PCR. Reverse transcription, fragmentation and cDNA labelling were conducted as suggested by the manufacture (Affymetrix). The processed samples were then hybridized to Affymetrix GeneChip *P. aeruginosa* Microarrays and the chips were washed, scanned and analysed according to the provided protocol (Affymetrix). The probe sets specific to strain PAO1 were filtered for statistically significant differences, signal above noise level, and a minimum twofold change using the statistics software MAS-5.0 from Affymetrix.

### Determination of β-galactosidase activity

β-galactosidase activity was measured in duplicates with three repeats starting with fresh LB cultures with appropriate antibiotics. Cultures were diluted 1:1000 into specified medium, supplemented with appropriate compounds as indicated when necessary. Unless otherwise stated, cells were grown at 37 °C for 4 hours before being assayed. The activity of β-galactosidase was quantified as described^38^. Results were plotted as average ± standard deviations.

### Measurement of MIC_50_, CC_50_ and EC_50_

The MIC_50_ of R101SPM was defined as the minimum inhibitory concentration resulting in a 50% inhibition of bacterial growth or *exsA* transcription. The growth of *P. aeruginosa* was monitored by the OD_600_ reading and the *exsA* transcription was reflected by the β-galactosidase activity of PCZ. Briefly, overnight PCZ cells were initially inoculated with 0.1% dilution in LB or in LB containing 0.7 mM spermidine, in presence or absence of serially diluted R101SPM. Cells were grown at 37 °C with 250 rpm shaking for recording the OD_600_ value and measuring the β-galactosidase activity at specified time points. In the case of MIC_50_ for TTSS inhibition of PCZ, we measured the beta-galactosidase activity by inoculating a larger volume of LB supplemented with 20 μM SPD. With a 4 hr culturing, cells were harvested by centrifugation and washed once with PBS and adjusted OD_600_ up to 1.0 with PBS before measuring the beta-galactosidase activity using described method. The CC_50_ (50% cytotoxic concentration) of R101SPM for cultured HeLa cells (ATCC: CCL-2) was determined as the concentration of compound that inhibits 50% of the conversion of 2-(2-methoxy-4-nitrophenyl)-3-(4-nitrophenyl)-5-(2,4-disulfophenyl)-2H- tetrazolium (WST-8) to formazan. Briefly, HeLa cells were seeded in 96-well plate at a density of 5000 cells per well in DMEM supplied with 10% FBS (Invitrogen). After overnight culturing, cells were washed once with PBS,incubated with FBS-free media, and exposed with aserial dilution of R101SPM. After exposure, the survival cells were washed once with PBS and assayed with the Cell Counting Kit-8 (Dojindo). The survival percentage of viable cells with R101SPM treatment compared to the number of untreated control cells was plotted against the R101SPM concentration. The 50% cytotoxic concentration (CC_50_) was analysed using the GraphPad Prism software (GraphPad Software, San Diego, CA). The EC_50_ (effective concentration) was calculated as the percentage of the WST-8 dehydrogenase activity present in the cells intoxicated with PAO1 with or without R101SPM compared to the activity in the intoxicated cells that was not treated with the inhibitor.

### Mouse infection

The protocol for mouse work was approved by the Institutional Animal Care and Use Committee (IACUC, Singapore) and conducted in accordance with the approved guidelines. Adult male FVB/N mice, 8–10 weeks old, were housed in Biological Resource Centre at Biopolis, Singapore. For preparation of bacterial cells for mouse infection, overnight culture of *P. aeruginosa* PAO1 was inoculated with a ratio of 1:200 into 10 ml LB medium and incubated at 37 °C for 4 hours with a 250 rpm shaking. With an OD_600_ of ~1.2, the cells were collected by centrifugation and washed with PBS for three times. For intraperitoneal infection, cells were mixed in PBS with various concentrations of R101SPM, and then 3 × 10^5^ cells were injected into mice following the approved protocol. Otherwise, cells were resuspended in R101SPM and 6 × 10^7^ cells were injected at one side of belly; simultaneously, different amounts of R101SPM dissolved in PBS were injected at the other side of belly. Differences in survival percentage were statistically analysed by a paired t test. CFU assay was conducted post of one day infection and normalized with the wet weight of livers as described elsewhere^39^.

### Chemistry

Chemicals commercially available were purchased from Sigma. Conjugation of Rhodamine with polyamine was catalysed by 1.1′-carbonyldiimidazole in dimethylformamide (DMF) solution unless otherwise stated, purified by HPLC, and verified by mass spectrometer and NMR analyses. Briefly, Rhodamine 101 (500 mg) was dissolved in DMF (150 ml) and mixed with 1,1′-carbonyldiimidazole (210 mg in 50 ml DMF). With a 30-min stirring at room temperature, the solution was added with spermine (880 mg in 5 ml DMF) and the reaction continued while stirring at room temperature. After 2 hours, the DMF solvent was then evaporated and the dried mixture was dissolved in 1M HCl solution. For purification, the crude product was subjected to HPLC on SymmetryPrep^TM^ C18 reverse-phase column, eluted with acetonitrile –water (gradient 20–40%) at a flow of 4 ml min^−1^. The general synthetic procedures and characterization of the compounds synthesized in this study were provided in Supplementary Method.

## Additional Information

**How to cite this article**: Wang, C. *et al*. Design and characterization of a polyamine derivative inhibiting the expression of type III secretion system in *Pseudomonas aeruginosa.* *Sci. Rep.* **6**, 30949; doi: 10.1038/srep30949 (2016).

## Supplementary Material

Supplementary Information
