# Supplementary material for: Design and characterization of a polyamine derivative inhibiting the expression of type III secretion system in Pseudomonas aeruginosa
Source: Sci Rep. 2016 Aug 3;6:30949. doi: 10.1038/srep30949 (PMC4971474; doi:10.1038/srep30949)
Supplement: Supplementary Information [file srep30949-s1.pdf]

SUPPLEMENTARY INFORMATION

For

The manuscript of **"Design and characterization of a polyamine derivative inhibiting the expression of type III secretion system in *Pseudomonas aeruginosa*"** by Chao Wang, Xiaoling Liu, Jing Wang, Zining Cui, Lian-Hui Zhang.

Summary

This Supplementary Data document contains three supplementary figures and three supplementary tables. Fig.S1 describes the synthesis of polyamine derivatives; Fig.S2 presents the protective effect of R101SPM on mice infected by P. aerguinosa, and Fig.S3 for the effect of R101SPM on other virulence factors of P. aerguinosa. Table S1 describes the ORFs whose transcriptions are upregulated by 5A treatment in the microarray analysis. Table S2 lists the ORFs whose transcriptions are decreased in response to 5A revealed in the microarray analysis. Table S3 lists the bacterial strains used in this study. The Supplementary method described the chemistry of synthesis and characterization of new compounds in this paper. The entire supplementary information is integrated into a PDF file.

Fig. S1

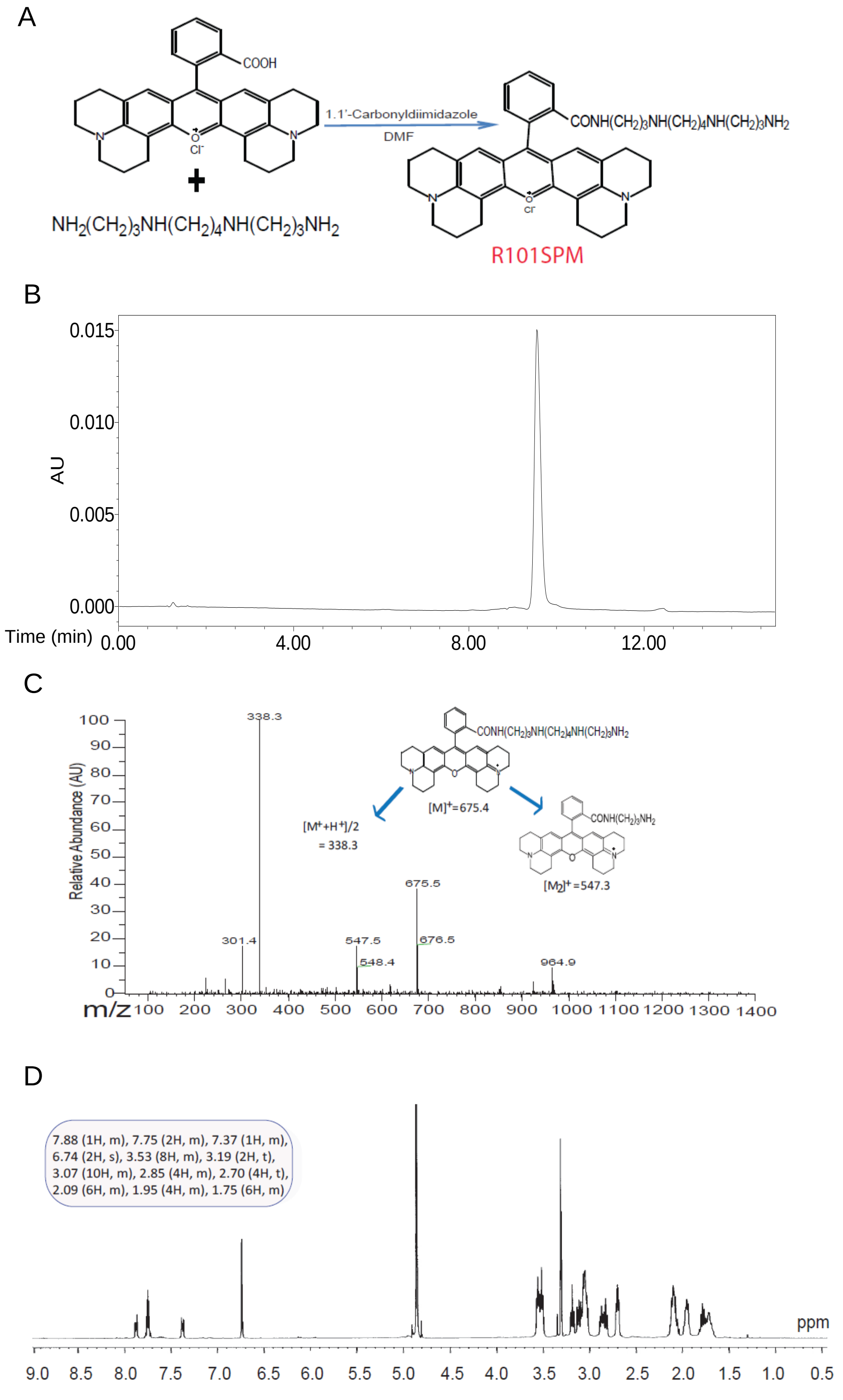
A
COOH
1.1'-Carbonyldiimidazole
DMF
CONH(CH2)3NH(CH2)4NH(CH2)3NH2
NH2(CH2)3NH(CH2)4NH(CH2)3NH2
R101SPM
B
0.015
0.010
0.005
0.000
AU
Time (min)
0.00
4.00
8.00
12.00
C
338.3
301.4
547.5
548.4
675.5
676.5
964.9
Relative Abundance (AU)
m/z
[M]+=675.4
[M++H+]/2
= 338.3
CONH(CH2)3NH2
[M2]+ =547.3
D
7.88 (1H, m), 7.75 (2H, m), 7.37 (1H, m), 6.74 (2H, s), 3.53 (8H, m), 3.19 (2H, t), 3.07 (10H, m), 2.85 (4H, m), 2.70 (4H, t), 2.09 (6H, m), 1.95 (4H, m), 1.75 (6H, m)
ppm

Fig. S1. Chemical synthesis of the spermidine derivative R101SPM.

(A) Schematic routine for chemical synthesis of R101SPM.

(B) HPLC profiles for the purifed R101SPM. 100 μmol of purified product was concentrated and resolved in 1M HCl aqueous solution, and then subjected to HPLC on the analytic C18 reverse-phase column (4.6 x 250mm, Waters), eluted with acetonitrile–water with a gradient of 20%-40% at a flow of 4 ml min^{-1}.

(C) Mass spectra of the synthesized R101SPM. The sample collected from HPLC separation was evaporated, dissolved in methanol–water (50:50 v/v), and then introduced into the mass spectrometer by loop injection. ESI-MS and tandem mass spectrometry were performed on a Finnigan LCQ DECA ion trap mass spectrometer.

(D) ^{1}H NMR spectra of the synthesized R101SPM. ^{1}H NMR spectra were recordedon a Bruker AMX400 (400 MHz) spectrometer using methanol-d4 solvent with tetramethylsilane as an internal standard at room temperature.

Fig.S2

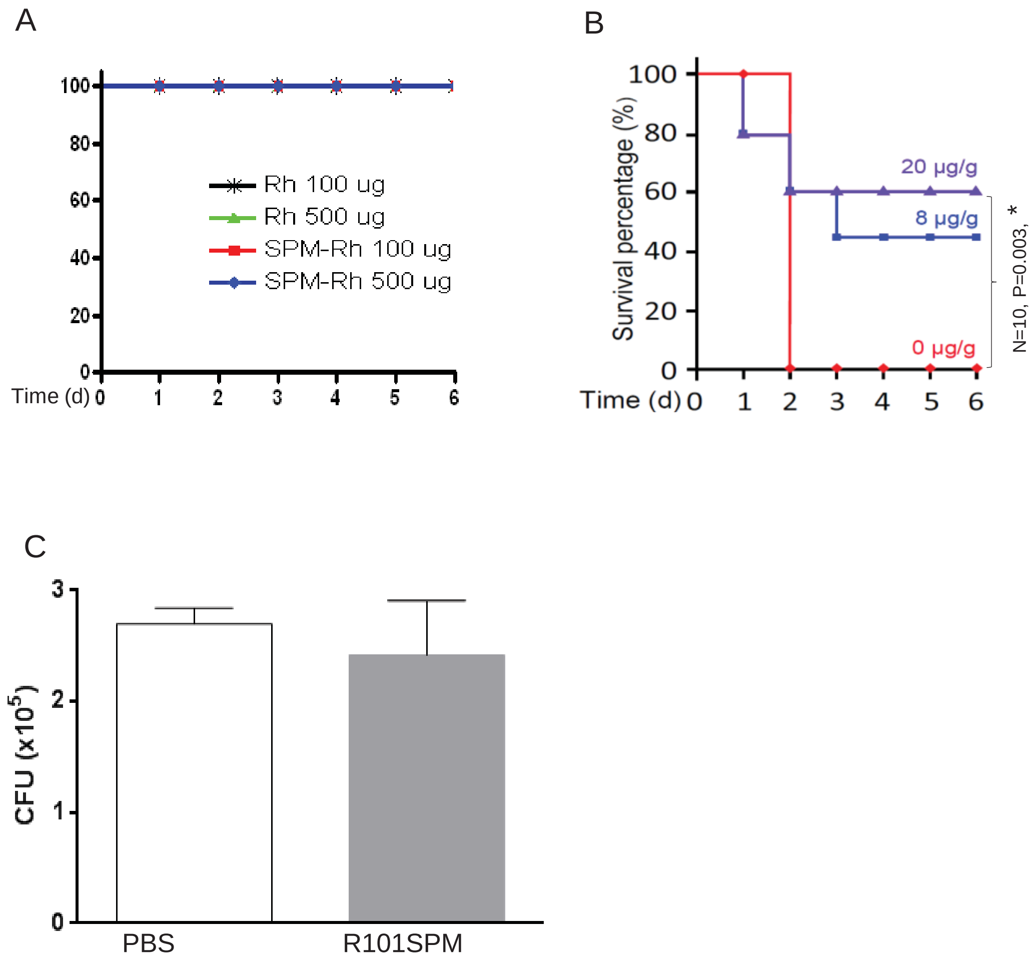

Fig. S2. Protective effect of R101SPM on mice infected by P. aeruginosa.

(A) The TTSS defective mutant P. aeruginosa PAO1(ΔexsA) was avirulent under the described conditions. Bacterial cells were prepared and mice injection were conducted as described for Fig. 5C. Five FVB/N male mice were included for each group.

(B) Protection of R101SPM for mice infected with *P. aeruginosa* by intraperitoneal injection. PAO1 cells were intraperitoneally infected at one side of belly, while R101SPM was intraperitoneally injected at the other side of belly.

(C) Viability assay of *P. aeruginosa* treated with 1mM R101SPM for half an hour.

Fig. S3

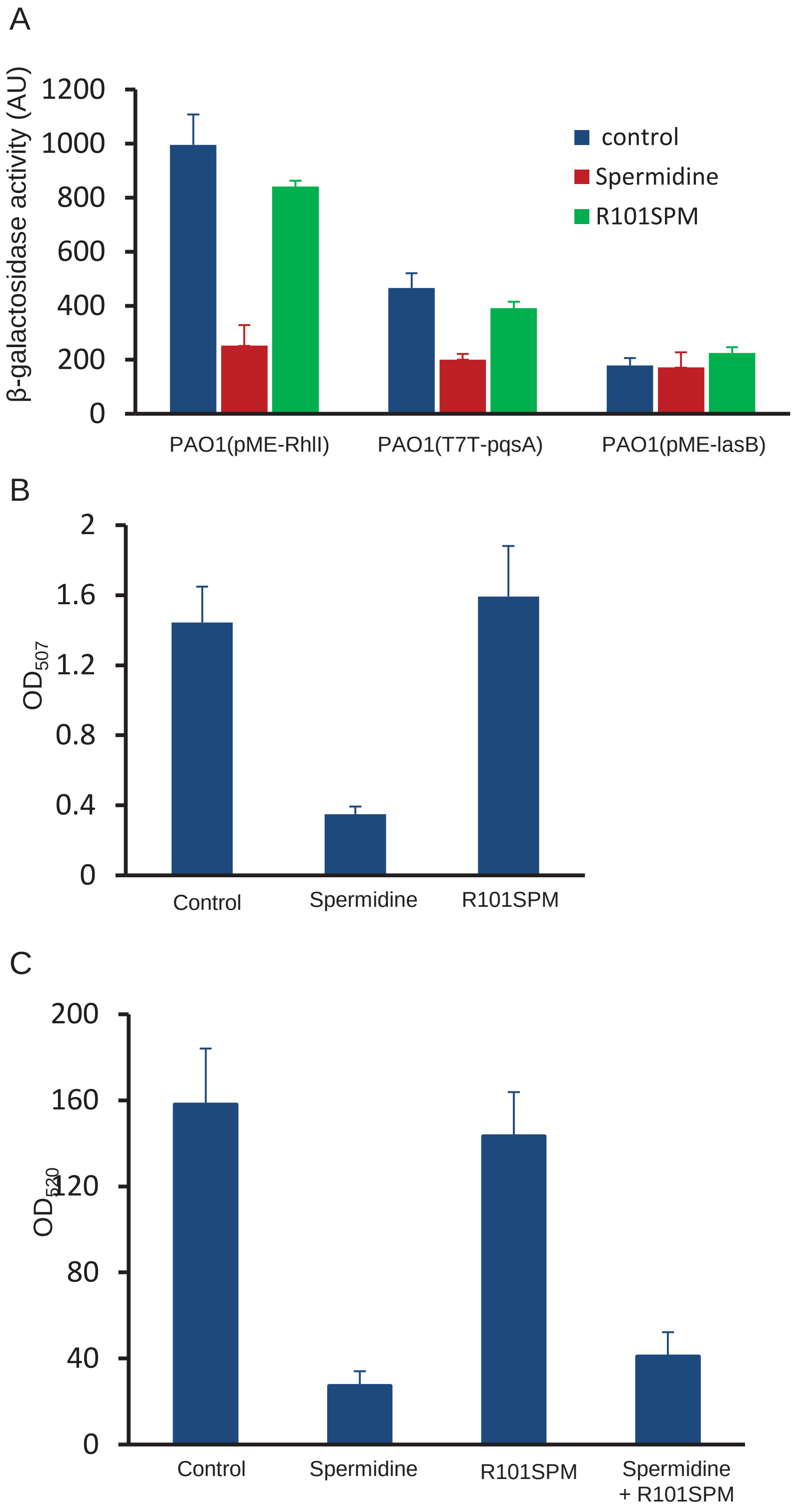
A
β-galactosidase activity (AU)
1200
1000
800
600
400
200
0
control
Spermidine
R101SPM
PAO1(pME-RhlI)
PAO1(T7T-pqsA)
PAO1(pME-lasB)
B
OD_{507}
2
1.6
1.2
0.8
0.4
0
Control
Spermidine
R101SPM
C
OD_{520}
200
160
120
80
40
0
Control
Spermidine
R101SPM
Spermidine + R101SPM

Fig. S3 Effect of R101SPM on other virulence factors of *P. aeruginosa*.

(A) Effect of R101SPM on quorum sensing system. Fresh specified bacterial strains were grown in LB medium (blue column) or supplied with 0.7mM spermidine (red column) or with 20 μM R101SPM (green column). At early stationary phase, the β-galactosidase activity was measured as described.

(B) Effects of R101SPM (20 μM) and spermidine (0.7 mM) biofilm formation. Overnight PAO1 cultures were diluted to OD_{600} of 0.03 in LB containing specified compounds, and duplicate 1ml aliquots were dispensed into 15ml falcon tubes. Following static incubation in a 30 °C chamber, the medium was removed, stained with 0.5% violent crystal dye and then washed gently with distilled water. For quantification, the blue dye was dissolved in 1ml ethanol for the OD_{507} reading.

(C) Effects of R101SPM (20 μM) and spermidine (0.7 mM) on pyocyanin production. Fresh PAO1 cells were inoculated with initial OD_{600} of 0.03 in the PA medium containing the specified compounds. After overnight growth at 37°C, pyocyanin was measured as described. Spd: spermidine; R101M: R101SPM; plus: presence of given compound(s), minus: absence of compounds.

Table S1. Upregulated genes with 5A treatment (>=2.0).

ORF[b]	Gene	fold[a]	Description
**Genes with known functions**			
PA0120		2.48	probable transcriptional regulator
PA0204		2.73	probable permease of ABC transporter
PA0212	mdcE	3.04	malonate decarboxylase gamma subunit
PA0229	pcaT	3.42	dicarboxylic acid transporter PcaT
PA0253	hudR	2.36	HudR
PA0268		2.32	probable transcriptional regulator
PA0293	aguB	2.52	N-carbamoylputrescine amidohydrolase
PA0382	micA	2.32	DNA mismatch repair protein MicA
PA0440	dypA	2.78	probable oxidoreductase
PA0478		2.67	probable N-acetyltransferase
PA0493		2.36	probable biotin-requiring enzyme
PA0517	nirC	3.36	probable c-type cytochrome precursor
PA0519	nirS	2.82	nitrite reductase precursor
PA0530		2.73	probable class III pyridoxal phosphate-dependent aminotransferase
PA0531		2.91	probable glutamine amidotransferase
PA0604	agtB	3.54	AgtB
PA0605	agtC	2.92	AgtC
PA0606	agtD	2.66	AgtD
PA0993	cupC2	3.54	chaperone CupC2
PA0996	pqsA	2.21	probable coenzyme A ligase
PA0997	pqsB	3.90	PqsB
PA0998	pqsC	3.29	PqsC
PA0999	pqsD	3.77	3-oxoacyl-[acyl-carrier-protein] synthase III
PA1000	pqsE	3.52	Quinolone signal response protein
PA1001	phnA	3.52	anthranilate synthase component I
PA1002	phnB	2.99	anthranilate synthase component II
PA1109		3.02	probable transcriptional regulator
PA1144		2.29	probable major facilitator superfamily (MFS) transporter
PA1169		2.68	probable lipoxygenase
PA1196		4.35	probable transcriptional regulator
PA1217		3.05	probable 2-isopropylmalate synthase
PA1275	cobD	2.92	cobalamin biosynthetic protein CobD
PA1303		2.31	signal peptidase
PA1347		3.71	probable transcriptional regulator
PA1389		2.23	probable glycosyl transferase
PA1556	ccoO2	2.46	Cytochrome c oxidase, cbb3-type, CcoO subunit
PA1596	htpG	2.62	heat shock protein HtpG
PA1617		2.68	probable AMP-binding enzyme
PA1637	kdpE	2.19	two-component response regulator KdpE

PA1660	hsiG2	2.40	HsiG2
PA1667	hsiJ2	2.65	HsiJ2
PA1779	nasA	2.78	assimilatory nitrate reductase
PA1845	tsi1	2.56	Tsi1
PA1867	xphA	2.23	XphA
PA1875		2.19	probable outer membrane protein precursor
PA1956	fapA	2.35	FapA
PA1962	azoR2	2.65	FMN-dependent NADH-azoreductase 2, AzoR2
PA2077		2.97	oleate 10S-lipoxygenase
PA2119	adh	2.13	alcohol dehydrogenase (Zn-dependent)
PA2124		2.54	probable dehydrogenase
PA2298		2.13	probable transcriptional regulator
PA2381		2.34	L-lactate dehydrogenase
PA2414		2.99	L-sorbosone dehydrogenase
PA2449		2.78	transcriptional regulator
PA2499	ykoA	2.74	probable deaminase
PA2922		2.58	probable hydrolase
PA2924	hisQ	2.22	histidine transport system permease HisQ
PA3045	rocA2	3.56	Two-component response regulator, RocA2
PA3126	ibpA	4.28	heat-shock protein IbpA
PA3232		2.87	probable nuclease
PA3337	rfaD	3.99	ADP-L-glycero-D-mannoheptose 6-epimerase
PA3507		2.76	probable short-chain dehydrogenase
PA3521	opmE	2.66	probable outer membrane protein precursor
PA3523		2.84	probable Resistance-Nodulation-Cell Division (RND) efflux membrane fusion protein precursor
PA3544	algE	3.03	Alginate production outer membrane protein AlgE precursor
PA3630		2.70	probable transcriptional regulator
PA3676	mexK	3.01	MexK
PA3677	mexJ	2.41	MexJ
PA3709		2.49	probable major facilitator superfamily (MFS) transporter
PA3713	spdH	2.36	spermidine dehydrogenase, SpdH
PA3761	nagE	2.96	N-Acetyl-D-Glucosamine phosphotransferase system transporter
PA3870	moaA1	2.35	molybdopterin biosynthetic protein A1
PA3957		2.69	probable short-chain dehydrogenase
PA4078		2.12	probable nonribosomal peptide synthetase
PA4082	cupB5	2.79	adhesive protein CupB5
PA4096	phlE	2.62	probable major facilitator superfamily (MFS) transporter
PA4127	hpcG	2.35	2-oxo-hept-3-ene-1,7-dioate hydratase
PA4150	acoA	3.09	probable dehydrogenase E1 component
PA4206	mexH	2.91	probable Resistance-Nodulation-Cell Division (RND) efflux membrane fusion protein precursor
PA4224	pchG	2.15	pyochelin biosynthetic protein PchG
PA4225	pchF	2.18	pyochelin synthetase
PA4303	tadZ	2.72	TadZ

PA4341		3.51	probable transcriptional regulator
PA4542	clpB	2.85	ClpB protein
PA4599	mexC	2.26	Resistance-Nodulation-Cell Division (RND) multidrug efflux membrane fusion protein MexC precursor
PA4759	dapB	2.50	dihydrodipicolinate reductase
PA4760	dnaJ	2.84	DnaJ protein
PA4761	dnaK	2.23	DnaK protein
PA4762	grpE	2.86	heat shock protein GrpE
PA4811	fdnH	2.77	nitrate-inducible formate dehydrogenase, beta subunit
PA5048		2.54	probable nuclease
PA5054	hslU	3.11	heat shock protein HslU
PA5098	hutH	2.21	histidine ammonia-lyase
PA5326	sphD	2.16	SphD
PA5353	glcF	2.16	glycolate oxidase subunit GlcF
PA5354	glcE	3.04	glycolate oxidase subunit GlcE
PA5375	betT1	2.61	BetT1
PA5418	soxA	3.44	sarcosine oxidase alpha subunit

Hypothetical proteins

PA0013	yhhQ	2.62	conserved hypothetical protein
PA0014		2.19	hypothetical protein
PA0069		2.39	conserved hypothetical protein
PA0097		2.25	hypothetical protein
PA0200		2.26	hypothetical protein
PA0213	mdcG	2.21	hypothetical protein
PA0442		3.43	hypothetical protein
PA0498		3.84	hypothetical protein
PA0698		2.86	hypothetical protein
PA0714		2.88	hypothetical protein
PA0717		3.44	hypothetical protein of bacteriophage Pf1
PA0990		2.66	conserved hypothetical protein
PA1168		2.64	hypothetical protein
PA1231		2.87	conserved hypothetical protein
PA1259		2.84	hypothetical protein
PA1360	ybiF	2.76	conserved hypothetical protein
PA1394		2.20	hypothetical protein
PA1426		2.10	hypothetical protein
PA1489		4.42	hypothetical protein
PA1531		4.35	hypothetical protein
PA1536		3.16	conserved hypothetical protein
PA1559		2.41	hypothetical protein
PA1560		2.91	hypothetical protein
PA1568		2.32	conserved hypothetical protein
PA1638	yneH	3.46	conserved hypothetical protein
PA1764		3.01	hypothetical protein

PA2002	atoE	3.22	conserved hypothetical protein
PA2036		3.42	hypothetical protein
PA2038		2.82	hypothetical protein
PA2072		2.47	conserved hypothetical protein
PA2144	glgP	2.98	hypothetical protein
PA2182		3.05	hypothetical protein
PA2357	msuE	3.15	hypothetical protein
PA2655		3.15	hypothetical protein
PA2753		3.24	hypothetical protein
PA2782		2.83	hypothetical protein
PA2792		2.95	hypothetical protein
PA2929		2.24	hypothetical protein
PA3402		2.94	hypothetical protein
PA3428		2.26	hypothetical protein
PA3905		2.86	hypothetical protein
PA3952		2.53	hypothetical protein
PA4106		2.55	conserved hypothetical protein
PA4134		2.48	hypothetical protein
PA4139		3.48	hypothetical protein
PA4713		2.24	hypothetical protein
PA4779		2.10	hypothetical protein
PA4823		2.46	hypothetical protein
PA4962	ybcI	3.18	conserved hypothetical protein
PA5136		2.33	hypothetical protein
PA5139		2.26	hypothetical protein
PA5475		2.99	hypothetical protein
PA5544		3.12	conserved hypothetical protein

a. Fold change represents the average change fold (log of base 2) of mRNA transcript levels in 5A-treated against untreated strains of *P. aeruginosa* PAO1.

b. Open reading frame (ORF) is as annotated in http://www.pseudomonas.com/.

Table S2 ORFs downregulated by SSA (>=1.5)

ORF[b]	Gene	fold[a]	Description
**Type III secretion system**			
PA1713	exsA	1.50	transcriptional regulator ExsA
PA0044	exoT	2.12	exoenzyme T
PA1705	pcrG	2.41	regulator in type III secretion
PA1706	pcrV	2.00	type III secretion protein PcrV
PA1707	pcrH	2.04	regulatory protein PcrH
PA1709	popD	1.88	Translocator outer membrane protein PopD precursor
PA1710	exsC	1.68	ExsC, exoenzyme S synthesis protein C precursor.
PA1714	exsD	1.66	ExsD
PA1715	pscB	1.95	type III export apparatus protein
PA1716	pscC	1.74	Type III secretion outer membrane protein PscC precursor
PA1717	pscD	1.93	type III export protein PscD
PA1718	pscE	1.97	type III export protein PscE
PA1719	pscF	1.76	type III export protein PscF
PA1720	pscG	2.03	type III export protein PscG
PA1721	pscH	1.83	type III export protein PscH
PA1722	pscI	1.80	type III export protein PscI
PA1723	pscJ	1.77	type III export protein PscJ
PA1724	pscK	1.52	type III export protein PscK
PA1725	pscL	1.70	type III export protein PscL
PA2808	ptrA	1.65	Pseudomonas type III repressor A
PA3841	exoS	1.86	exoenzyme S
**Other functions**			
PA0031	betC	2.64	choline sulfatase
PA0058	dsbM	1.71	DsbM
PA0129	bauD	1.57	Amino acid permease
PA0138		2.74	probable permease of ABC transporter
PA0164		2.48	probable gamma-glutamyltranspeptidase
PA0182	yjgI	3.03	probable short-chain dehydrogenase
PA0208	mdcA	2.65	malonate decarboxylase alpha subunit
PA0220		2.39	amino acid APC family transporter
PA0247	pobA	2.03	p-hydroxybenzoate hydroxylase
PA0352	yicE	2.75	probable transporter
PA0364		1.83	probable oxidoreductase
PA0450		1.64	probable phosphate transporter
PA0489		1.80	probable phosphoribosyl transferase
PA0508		1.62	probable acyl-CoA dehydrogenase
PA0513	nirG	2.40	NirG
PA0514	nirL	1.54	heme d1 biosynthesis protein NirL
PA0524	norB	2.48	nitric-oxide reductase subunit B

PA0534	pauB1	2.50	FAD-dependent oxidoreductase
PA0641		1.89	probable bacteriophage protein
PA0690	pdtA	2.52	PdtA
PA0707	toxR	1.72	transcriptional regulator ToxR
PA0845	cerN	2.91	CerN
PA0866	aroP2	2.08	aromatic amino acid transport protein AroP2
PA0880		1.52	probable ring-cleaving dioxygenase
PA0884	dctP	2.58	probable C4-dicarboxylate-binding periplasmic protein
PA1003	mvfR	1.55	Transcriptional regulator MvfR
PA1127	gsp69	2.30	probable oxidoreductase
PA1141		2.50	probable transcriptional regulator
PA1326	ilvA2	1.52	threonine dehydratase, biosynthetic
PA1384	galE	2.28	UDP-glucose 4-epimerase
PA1385		2.18	probable glycosyl transferase
PA1519		1.61	probable transporter
PA1537		2.09	probable short-chain dehydrogenase
PA1565	pauB2	2.43	FAD-dependent oxidoreductase
PA1566	pauA3	2.06	Glutamylpolyamine synthetase
PA1651		1.72	probable transporter
PA1785	nasT	1.95	NasT
PA1820	nhaB	3.19	sodium/proton antiporter NhaB
PA1869		1.97	probable acyl carrier protein
PA1908		1.72	probable major facilitator superfamily (MFS) transporter
PA1919	nrdG	2.99	class III (anaerobic) ribonucleoside-triphosphate reductase activating protein
PA1947	rbsA	1.53	ribose transport protein RbsA
PA1978	erbR	1.96	response regulator ErbR
PA1980	eraR	1.59	response regulator EraR
PA1983	exaB	2.55	cytochrome c550
PA2005		3.38	probable transcriptional regulator
PA2018	mexY	1.59	Resistance-Nodulation-Cell Division (RND) multidrug efflux transporter MexY
PA2051		3.35	probable transmembrane sensor
PA2073		1.74	probable transporter (membrane subunit)
PA2187		1.67	probable alcohol dehydrogenase (Zn-dependent)
PA2190		2.34	adenylate cyclase ExoY
PA2201		1.56	probable amino acid permease
PA2216		1.96	probable aldehyde dehydrogenase
PA2275	yahK	1.53	probable transcriptional regulator
PA2278	arsB	1.81	ArsC protein
PA2355	msuC	2.60	methanesulfonate sulfonatase MsuD
PA2385	pvdQ	1.78	L-ornithine N5-oxygenase
PA2388	fpvR	1.88	PvdR
PA2391	opmQ	1.54	PvdP
PA2392	pvdP	2.23	probable dipeptidase precursor
PA2394	pvdN	1.81	PvdO
PA2396	pvdF	1.99	pyoverdine biosynthesis protein PvdE

PA2413	pvdH	1.93	L-2,4-diaminobutyrate:2-ketoglutarate 4-aminotransferase, PvdH
PA2432	bexR	1.54	bistable expression regulator, BexR
PA2444	glyA2	1.63	serine hydroxymethyltransferase
PA2473		1.83	probable glutathione S-transferase
PA2494	mexF	3.19	Resistance-Nodulation-Cell Division (RND) multidrug efflux transporter MexF
PA2512	antA	1.53	anthranilate dioxygenase large subunit
PA2513	antB	1.59	anthranilate dioxygenase small subunit
PA2516	xylZ	2.06	toluate 1,2-dioxygenase electron transfer component
PA2520	czcA	1.61	Resistance-Nodulation-Cell Division (RND) divalent metal cation efflux transporter Czc
PA2522	czcC	1.66	outer membrane protein precursor CzcC
PA2547		3.41	probable transcriptional regulator
PA2601		1.63	probable transcriptional regulator
PA2838		3.89	probable transcriptional regulator
PA2917		2.23	probable transcriptional regulator
PA3053		3.24	probable hydrolytic enzyme
PA3095	xcpZ	2.10	general secretion pathway protein M
PA3122	yibL	1.92	probable transcriptional regulator
PA3175		1.69	probable arginase family protein
PA3188	gltG	3.06	probable permease of ABC sugar transporter
PA3279	oprP	1.85	Phosphate-specific outer membrane porin OprP precursor
PA3319	plcN	2.45	non-hemolytic phospholipase C precursor
PA3395	nosY	1.98	NosY protein
PA3467		2.45	probable major facilitator superfamily (MFS) transporter
PA3541	alg8	2.07	alginate biosynthesis protein Alg8
PA3548	algI	3.39	alginate o-acetyltransferase AlgI
PA3609	potC	2.89	polyamine transport protein PotC
PA3776		3.93	probable transcriptional regulator
PA3842	spcS	2.17	specific Pseudomonas chaperone for ExoS, SpcS
PA3909	eddB	2.48	Extracelullar DNA degradation protein, EddB
PA4083	cupB4	2.80	chaperone CupB4
PA4091	hpaA	2.09	4-hydroxyphenylacetate 3-monooxygenase large chain
PA4092	hpaC	2.02	4-hydroxyphenylacetate 3-monooxygenase small chain
PA4130		1.84	probable sulfite or nitrite reductase
PA4143	cyaB; cvaB	2.04	probable toxin transporter
PA4306	flp	2.76	Type IVb pilin, Flp
PA4330		1.50	probable enoyl-CoA hydratase/isomerase
PA4343		1.80	probable major facilitator superfamily (MFS) transporter
PA4363	iciA	1.69	inhibitor of chromosome initiation IciA
PA4827	nat	3.81	arylamine N-acetyltransferase
PA4859		1.50	probable permease of ABC transporter
PA4860		3.08	probable permease of ABC transporter
PA4867	ureB	2.77	urease beta subunit
PA4888	desB	1.58	acyl-CoA delta-9-desaturase, DesB
PA4891	ureE	1.76	urease accessory protein UreE

PA4898	opdK	3.13	histidine porin OpdK
PA4899		2.10	probable aldehyde dehydrogenase
PA4979		2.40	probable acyl-CoA dehydrogenase
PA4986		2.56	probable oxidoreductase
PA4990		4.01	SMR multidrug efflux transporter
PA5075		2.25	probable permease of ABC transporter
PA5097	hutT	2.31	probable amino acid permease
PA5150		1.69	probable short-chain dehydrogenase
PA5293		1.63	probable transcriptional regulator
PA5311		1.50	probable major facilitator superfamily (MFS) transporter
PA5328	sphB	2.10	SphB
PA5398	dgcA	1.79	DgcA, Dimethylglycine catabolism
PA5417	soxD	2.94	sarcosine oxidase delta subunit
PA5479	gltP	3.03	proton-glutamate symporter
**Hypothetical proteins**			
PA0022	yrdC	2.74	conserved hypothetical protein
PA0234		1.59	hypothetical protein
PA0278		1.70	hypothetical protein
PA0311		1.97	hypothetical protein
PA0327		1.78	hypothetical protein
PA0496	ybgK	2.60	conserved hypothetical protein
PA0540		2.39	hypothetical protein
PA0695		2.16	hypothetical protein
PA0696		2.03	hypothetical protein
PA0702		1.74	hypothetical protein
PA0735		2.32	hypothetical protein
PA0775	yecO	1.66	conserved hypothetical protein
PA0824		2.51	hypothetical protein
PA0834		1.61	conserved hypothetical protein
PA0915	yehS	2.76	conserved hypothetical protein
PA0981		2.95	hypothetical protein
PA1126		1.61	hypothetical protein
PA1134		1.54	hypothetical protein
PA1190	yohC	1.78	conserved hypothetical protein
PA1200		1.71	conserved hypothetical protein
PA1211		3.45	hypothetical protein
PA1214		1.66	hypothetical protein
PA1346		2.15	hypothetical protein
PA1433		2.41	conserved hypothetical protein
PA1499		2.02	conserved hypothetical protein
PA1503		1.84	hypothetical protein
PA1841		1.58	hypothetical protein
PA1878		1.68	hypothetical protein
PA1891		1.68	hypothetical protein

PA1893		1.96	hypothetical protein
PA1917		2.03	hypothetical protein
PA1929		1.73	hypothetical protein
PA2043		3.06	hypothetical protein
PA2122		1.73	hypothetical protein
PA2206		3.27	hypothetical protein
PA2210		1.53	conserved hypothetical protein
PA2376		2.28	hypothetical protein
PA2421		2.06	hypothetical protein
PA2427		2.17	hypothetical protein
PA2448		2.00	hypothetical protein
PA2490	ydbB	2.11	conserved hypothetical protein
PA2498	yahD	1.58	conserved hypothetical protein
PA2566		2.80	conserved hypothetical protein
PA2602		2.59	hypothetical protein
PA2628		1.53	hypothetical protein
PA2752	yqaA	2.03	conserved hypothetical protein
PA2916		2.01	hypothetical protein
PA3281		1.56	hypothetical protein
PA3283		1.93	conserved hypothetical protein
PA3284		2.23	hypothetical protein
PA3320		3.22	hypothetical protein
PA3380	phnG	1.53	conserved hypothetical protein
PA3425		1.85	hypothetical protein
PA3470		1.55	hypothetical protein
PA3518		2.21	hypothetical protein
PA3519		1.53	hypothetical protein
PA3843		2.37	hypothetical protein
PA3991		2.64	hypothetical protein
PA4287		1.62	hypothetical protein
PA4582		2.02	conserved hypothetical protein
PA4583	rtcB	2.72	conserved hypothetical protein
PA4584	ycgL	1.76	conserved hypothetical protein
PA4794		1.95	hypothetical protein
PA4818		1.57	conserved hypothetical protein
PA4882		1.82	hypothetical protein
PA4985		3.95	hypothetical protein
PA5194		2.08	hypothetical protein
PA5480		2.64	hypothetical protein
PA5509		1.65	hypothetical protein

a. Fold change represents the average change fold (log of base 2) of mRNA transcript levels in 5A-treated against untreated strains of *P. aeruginosa* PAO1.

b. Open reading frame (ORF) is as annotated in http://www.pseudomonas.com/.

Table S3. Strains and plasmids used in this study*

Strain or plasmid	Description	Source or reference
E. coli		
DH5α	F– f80 d*lacZ*DM15 *endA1hsdR17* (r_k^- m_k^-) *supE44 thi-1 gyrA96* D(*lacZYA-argF*), used for plasmid transformation	Gibco
S17-1	res^- pro mod^+ integrated copy of RP4, mob^+, used for incorporating constructs into *P. aeruginosa*	Laboratory collection
**Plasmids**		
Tn7T-Gm-lacZ	mini-Tn7T cloning and delivery vector for lacZ transcriptional fusion vector, Gm^r	(1)
pFLP2	Source of Flp recombinase; Cb^r	(1)
Tn7-pqsA	mini-Tn7T-Gm-lacZ carrying the promoter region of pqsA for reporting the pqsA transcription	(2)
pME6016	shuttle vector for transcriptional lacZ fusion in P.aeruginosa, Tc^r	(3)
pME-RhlI	pME6016 carrying the promoter region of rhlI for reporting the rhlI transcription, Tc^r	(2)
pME-lasB	pME6016 carrying the promoter region of lasB for reporting the lasB transcription, Tc^r	(2)
pEX18Gm	Suicide plasmid carrying *sacBR*, Gm^r	(4)
pEX18G-vfr	pEX18Gm containing the vfr flanking region with part of vfr being deleted in frame, Gm^r	This study
pEX18G-spuB	pEX18Gm containing the spuB flanking region with part of spuB being deleted in frame, Gm^r	This study
pEX18G-spuE	pEX18Gm containing the spuE flanking region with part of spuE being deleted in frame, Gm^r	This study
P. aeruginosa		
PAO1	Prototrophic laboratory strain	Laboratory collection
PCZ	PAO1 carrying promoterless lacZ fused to the exsCEBA promoter and integrated at the attB site of the PAO1 chromosome, Tc^r	(5)
PAO1(ΔexsA)	PAO1 with inactivation of exsA, Gm^r	(5)
PCZ (Δvfr)	PCZ with inframe deletion of vfr, Tc^r	This study
PCZ (ΔspuB)	PCZ with inframe deletion of spuB, Tc^r	This study
PCZ (ΔspuE)	PCZ with inframe deletion of spuE, Tc^r	This study
PAO1(pME-rhlI)	PAO1 carrying the pME-rhlI plasmid for reporting the rhlI transcription, Tc^r	(2)
PAO1(pME-lasB)	PAO1 carrying the pME-lasB plasmid for reporting the lasB transcription, Tc^r	(2)
PAO1(T7T-pqsA)	PAO1 chromosomally integrated at its *att* site with a fragment in which the promoter region of pqsA was transcriptionally fused with the promoteless lacZ gene for reporting the pqsA transcription.	(2)

* Symbol: Gm^r, gentamicin resistant; Cb^r, carbenicillin resistant; Tc^r, tetracycline resistant.

Reference

1. **Choi KH, Schweizer HP.** 2006. mini-Tn7 insertion in bacteria with secondary, non-glmS-linked attTn7 sites: example Proteus mirabilis HI4320. Nat Protoc **1:**170-178.
2. **Lee J, Wu J, Deng Y, Wang J, Wang C, Chang C, Dong Y, Williams P, Zhang LH.** 2013. A cell-cell communication signal integrates quorum sensing and stress response. Nature chemical biology **9:**339-343.
3. **Schnider-Keel U, Seematter A, Maurhofer M, Blumer C, Duffy B, Gigot-Bonnefoy C, Reimmann C, Notz R, Defago G, Haas D, Keel C.** 2000. Autoinduction of 2,4-diacetylphloroglucinol biosynthesis in the biocontrol agent Pseudomonas fluorescens CHA0 and repression by the bacterial metabolites salicylate and pyoluteorin. J Bacteriol **182:**1215-1225.
4. **Hoang TT, Karkhoff-Schweizer RR, Kutchma AJ, Schweizer HP.** 1998. A broad-host-range Flp-FRT recombination system for site-specific excision of chromosomally-located DNA sequences: application for isolation of unmarked Pseudomonas aeruginosa mutants. Gene **212:**77-86.
5. **Zhou L, Wang J, Zhang LH.** 2007. Modulation of bacterial Type III secretion system by a spermidine transporter dependent signaling pathway. PLoS One **2:**e1291.

Supplementary method

General synthetic procedure

Chemicals commercially available were purchased from Sigma. Conjugation of Rhodamines or other reagents with polyamines was catalysed by 1,1-carbonyldiimidazole unless otherwise stated, purified by HPLC and verified by MS or NMR analyses. Synthesis of R101SPM in detail: Rhodamine 101 500 mg was dissolved in DMF 150 ml and a solution of 1,1-carbonyldiimidazole 210 mg in DMF 50 ml was added with stirring at room temperature for 30 min., the mixture was added with a solution of spermine 880 mg in DMF 5 ml and continued stirring at room temperature for 2 hours, the solvent DMF was then evaporated and the crude product was acidized with 1M HCl solution, and then subjected to HPLC on SymmetryPrep™ C18 reverse-phase column (7 μm, 7.8 x 150mm, Waters), eluted with acetonitrile-water (gradient 20%-40%) at a flow of 4 ml/min. For the synthesis of R101SPD1 or R101SPD2, two types of Boc-protected spermidines were chosen as polyamines. The deprotected condition was TFA in CH_2Cl_2 at room temperature for 2 hr.

RhBSPM: ESI (M^+): 627.5/627.4 (Det./Cal.).

BenzoylbenzeneSPM: ESI ($M+H^+$): 412.3/411.3 (Det./Cal.).

AcidblueSPM: ESI (M^+): 852.5/852.4 (Det./Cal.).

R110SPM: H^1-NMR (D_2O): δ 7.85 (2H), 7.77 (1H), 7.34 (2H), 7.14 (3H), 7.04 (2H), 3.28 (2H), 2.74 (10H), 1.91 (4H), 1.71 (4H).

FluorenoneSPM: H^1-NMR (D_6-MeOD): δ 7.82 (m, 1H), 7.73 (m, 1H), 7.63 (m, 3H), 7.50 (m, 1H), 7.39 (m, 1H), 3.58 (t, 2H), 3.20 (m, 10H), 1.06 (m, 4H), 1.86 (m, 4H). C^{13}-NMR: δ 194.9, 170.8, 146.8, 145.1, 137.1, 136.7, 135.4, 134.7, 131.5, 131.1, 129.9, 125.6, 123.8, 122.2, 48.5, 48.4, 46.4, 46.0, 37.9, 37.3, 27.8, 25.5, 24.5, 24.4.

BiotinSPM: ESI ($M+H^+$): 429.3/428.3 (Det./Cal.); H^1-NMR (D_4-MeOD): δ 4.35 (m, 1H), 4.15 (m, 1H), 2.90 (m, 16H), 1.62 (m, 15H); C^{13}-NMR: δ 177.4, 166.2, 63.6, 61.8, 57.3, 48.4, 48.3, 46.5, 46.0, 41.2, 38.0, 37.0, 36.6, 30.0, 29.7, 27.8, 26.8, 25.5, 24.42, 24.40.

CinnamincSPM: ESI ($M+H^+$): 393.2/392.3 (Det./Cal.); H^1-NMR (D_4-MeOD): δ 7.51 (d, J = 12 Hz, 1H) , 7.16 (m, 2H), 7.13 (d, 1H), 6.50 (d, J = 12 Hz, 1H),3.87 (s, 6H), 3.44 (t,.2H), 3.10 (m, 10H),2.09 (m, 2H), 1.95 (m, 6H); C^{13}-NMR: δ 168, 151, 149, 141, 128, 122, 117, 111, 110, 55, 47, 46, 45, 44, 36, 35, 27, 24, 23.

AcridineSPM: ESI ($M+H^+$): 444.2/443.2 (Det./Cal.); H^1-NMR (D_4-MeOD): δ 8.39 (d, J = 8Hz, 1H), 7.76 (s, 1H), 7.73 (s, 1H), 7.69 (d, J = 12 Hz, 1H), 7.59, (m, 1H), 7.40 (m, 1H), 3.91 (s, 3H), 3.10 (m, 10H), 2.30 (m, 2H), 1.98 (m, 2H), 1.70 (m, 4H); C^{13}-NMR: δ 163.4, 163.0, 158.6, 158.3, 142.1, 129.5, 129.1, 125.4, 121.7, 118.8, 116.0, 111.6, 104.0, 56.8, 48.34, 48.32, 47.4, 46.4, 45.9, 27.5, 25.5, 24.35, 24.31.

R101SPM：ESI (M^+): 675.5/675.4 (Det./Cal.); H^1-NMR (D_4-MeOD): δ 7.88 (m, 1H), 7.75 (m, 2H), 7.37 (m, 1H), 6.74 (s, 2H), 3.53 (m, 8H), 3.19 (t, 2H), 3.07 (m, 10H), 2.85 (m, 4H), 2.70 (t, 4H), 2.09 (m, 6H), 1.95 (m, 4H), 1.75 (m, 6H); C^{13}-NMR: δ 170.9, 156.8, 153.6, 152.6, 137.5, 133.7, 132.2, 132.1, 131.3, 129.2, 127.6, 125.3, 114.2, 106.7, 52.0, 51.5, 48.3, 48.2, 46.5, 46.0, 37.9, 37.6, 28.7, 27.6, 25.5, 24.4, 22.0, 21.1, 21.0.

R101SPD1：ESI (M^+): 618.5/618.4 (Det./Cal.); H^1-NMR (D_6-DMSO): δ 7.73 (m, 1H), 7.47 (m, 2H), 6.95 (m, 1H), 6.00 (s, 2H), 3.06 (m, 8H), 2.78 (m, 12H), 2.46 (m, 4H), 1.93 (m, 4H), 1.76 (m, 4H), 1.50 (m, 6H) ;C^{13}-NMR: δ 169.2, 155.2, 148.9, 144.8, 134.2, 131.0, 129.5, 125.0, 124.9, 124.5, 123.6, 118.4, 108.4, 106.0, 50.4, 49.9, 47.4, 46.0, 39.5, 38.4, 27.9, 25.9, 25.4, 23.9, 22.7, 22.1, 22.0.

R101SPD2: ESI (M^+): 618.5/618.4 (Det./Cal.); H^1-NMR (D_2O): 7.73 (m, 3H), 7.15 (d, 1H), 6.75 (s, 2H), 3.45 (m, 8H), 3.00 (m, 10H), 2.67 (m, 6H), 1.96 (m, 10H), 1.46 (m, 2H), 1.22 (m, 2H); C^{13}-NMR: δ 171.0, 152.0, 151.0, 149.0, 136.0, 130.9, 130.7, 130.2, 130.1, 127.5, 126.0, 124.0, 112.0, 105.0, 50.5, 50.1, 47.2, 44.3, 38.6, 36.5, 27.0, 25.4, 23.7, 22.7, 20.2, 19.5, 19.2.

R101PYA: ESI (M^+): 673.5/673.4 (Det./Cal.); H^1-NMR (D_4-MeOD): δ 7.90 (m, 1H), 7.75 (m, 2H), 7.37 (m, 1H), 6.73 (s, 2H), 3.53 (m, 8H), 3.04 (m, 24H), 1.97 (m, 12H); C^{13}-NMR: δ 169.0, 155.5, 152.0, 151.0, 135.8, 132.1, 130.9, 130.7, 129.7, 127.5, 126.0, 123.8, 113.7, 105.0, 54.0, 51.0, 50.5, 49.9, 49.5, 37.5, 36.2, 33.8, 27.2, 26.2, 25.2, 20.5, 19.5, 19.4.

R101BPA: ESI (M^+): 604.5/604.4 (Det./Cal.); H^1-NMR (D_4-MeOD): δ 7.87 （m， 1H）, 7.74 (m, 2H), 7.37 (m, 1H), 6.74 (s, 2H), 3.53 (m, 8H), 3.19 (t, 2H), 3.07 (m, 4H), 2.96 (m, 4H), 2.85 (t, 2H), 2.70 (t, 4H), 2.10 (m, 4H), 1.96 (m, 6H), 1.79 (m, 2H); C^{13}-NMR: δ 170.9, 156.7, 153.6, 152.6, 137.6, 133.7, 132.2, 132.1, 131.2, 129.2, 127.6, 125.4, 114.2, 106.5, 52.0, 51.5, 46.7, 46.0, 37.9, 37.6, 28.7, 27.6, 25.5, 22.0, 21.1, 21.0.

R101PUT: ESI (M^+): 561.5/561.3 (Det./Cal.); H^1-NMR (D_4-MeOD): δ 7.84 (m, 1H), 7.74 (m, 2H), 7.37 (m, 1H), 6.74 (s, 2H), 3.55 (m, 8H), 3.08 (m, 6H), 2.83 (m, 2H), 2.70 (m, 4H), 2.10 (m, 4H), 1.95 (m, 4H), 1.50 (m, 4H); C^{13}-NMR: δ 173.0, 161.3, 152.1, 151.0, 136.5, 132.2, 130.7, 129.7, 127.6, 126.4, 124.1, 123.9, 112.9, 105.2, 50.5, 50.0, 38.9, 38.4, 27.4, 26.1, 24.5, 20.5, 19.7, 19.5.

R101PRA: ESI (M^+): 547.5/547.3 (Det./Cal.); H^1-NMR (D_4-MeOD): δ 7.86 (m, 1H), 7.50 (m, 2H), 7.38 (m, 1H), 6.74 (s, 2H), 3.53 (m, 8H), 3.18 (t, 2H), 3.07 (t, 4H), 2.72 (m 6H), 2.10 (m, 4H), 1.95 (m, 4H), 1.72 (m, 2H); C^{13}-NMR: δ 170.9, 156.8, 153.7, 152.6, 137.6, 133.8, 132.2, 132.1, 131.3, 129.1, 127.6, 125.3, 114.5, 106.8, 52.0, 51.5, 38.0, 37.5, 28.9, 28.7, 22.0, 21.0, 20.9.
